# Antigen receptor-mediated depletion of FOXP3 in induced regulatory T-lymphocytes via PTPN2 and FOXO1

**DOI:** 10.1038/ncomms9576

**Published:** 2015-10-13

**Authors:** Evita Bothur, Hartmann Raifer, Claudia Haftmann, Anna-Barbara Stittrich, Anne Brüstle, Dirk Brenner, Nadine Bollig, Maria Bieringer, Chol-Ho Kang, Katharina Reinhard, Bärbel Camara, Magdalena Huber, Alexander Visekruna, Ulrich Steinhoff, Antje Repenning, Uta-Maria Bauer, Veronika Sexl, Andreas Radbruch, Tim Sparwasser, Mir-Farzin Mashreghi, Tak Wah Mak, Michael Lohoff

**Affiliations:** 1 Institute for Medical Microbiology and Hygiene, University of Marburg, Hans Meerwein Strasse 2, 35037 Marburg, Germany; 2 German Rheumatism Research Center Berlin, Charitéplatz 1, 10117 Berlin, Germany; 3 The John Curtin School of Medical Research, The Australian National University, GPO Box 334, Canberra City, ACT 2600, Australia; 4 Experimental and Molecular Immunology, Luxembourg Institute of Health, 29 rue Henri Koch, L-4354 Esch-sur-Alzette, Luxembourg; 5 Odense Research Center for Anaphylaxis (ORCA), Department of Dermatology and Allergy Center, Odense University Hospital, University of Southern Denmark, Odense, Denmark; 6 Institute of Molecular Biology and Tumor Research, University of Marburg, Emil-Mannkopff-Straße 2, 35032 Marburg, Germany; 7 Institute for Pharmacology and Toxicology, University of Veterinary Medicine Vienna, Veterinärplatz 1, A-1210 Wien, Austria; 8 Institute of Infection Immunology, TWINCORE, Feodor-Lynen-Straße 7, 30625 Hannover, Germany

## Abstract

Regulatory T-cells induced via IL-2 and TGFβ *in vitro* (iTreg) suppress immune cells and are potential therapeutics during autoimmunity. However, several reports described their re-differentiation into pathogenic cells *in vivo* and loss of their key functional transcription factor (TF) FOXP3 after T-cell antigen receptor (TCR)-signalling *in vitro*. Here, we show that TCR-activation antagonizes two necessary TFs for *foxp3* gene transcription, which are themselves regulated by phosphorylation. Although the tyrosine phosphatase PTPN2 is induced to restrain IL-2-mediated phosphorylation of the TF STAT5, expression of the TF FOXO1 is downregulated and miR-182, a suppressor of FOXO1 expression, is upregulated. TGFβ counteracts the FOXP3-depleting TCR-signal by reassuring FOXO1 expression and by re-licensing STAT5 phosphorylation. Overexpressed phosphorylation-independent active versions of FOXO1 and STAT5 or knockdown of PTPN2 restores FOXP3 expression despite TCR-signal and absence of TGFβ. This study suggests novel targets for stabilisation and less dangerous application of iTreg during devastating inflammation.

## Abstract (web summary)

Antigen stimulation *in vivo* can reprogram T regulatory cells to lose the expression of Foxp3 and become effector cells. Here the authors show that the mechanism involves dephosphorylation of STAT5 by PTPN2 and downregulation of Foxo1 by miR-182.

## Graphical abstract

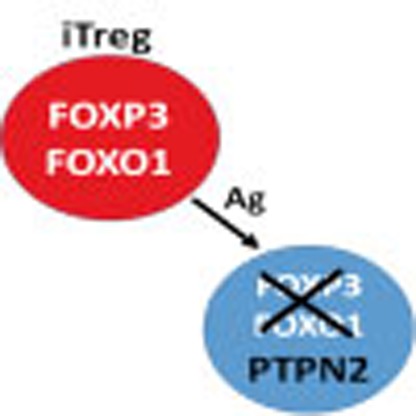

Regulatory T-cells (Tregs) suppress immune functions of effector cells^1^ and have been divided into subsets, among which tTregs and pTregs (together previously termed nTreg) are the most important ones^2^. Although tTregs develop in the thymus and are the prevailing subset of Tregs found in the periphery, pTregs are considered to develop from conventional CD4^+^ T-cells primarily at mucosal sites^3^,^4^ and cells with many similarities to pTreg termed iTregs can be generated *in vitro* from conventional CD4^+^ T-cells by stimulation via the T-cell receptor (TCR) in the presence of the cytokines IL-2 and TGFβ (ref. 5). Development and function of Tregs have been tightly linked to the transcription factor FOXP3, since its mutation leads to an autoimmune syndrome^6^,^7^,^8^ and overexpression of FOXP3 in conventional T-cells induces the majority of genes characteristic for a Treg signature^8^,^9^,^10^.

Apart from its promoter, three conserved enhancer regions, termed CNS1 to 3, have been implicated in regulation of FOXP3 expression and Treg development^11^. CNS3 is particularly relevant for generating tTregs through binding of the TF c-Rel^11^,^12^,^13^,^14^, while CNS1 controls development of pTregs^11^. CNS1-deficient mice develop autoimmunity specifically at mucosal sites where pTregs are particularly located^3^. CNS2 controls the stability of FOXP3 expression by changes in the methylation status of CpG motifs^15^,^16^. In particular, stable demethylation of this locus in tTregs correlates with continuous FOXP3 expression, while ongoing methylation in iTregs or pTregs indicates decay of FOXP3 expression after removal of TGFβ (ref. 15).

Several other transcription factors also contribute to FOXP3 expression. For CNS1, these include the TFs Smad3 and NFAT and reflect TGFβ activity^17^, while the TFs CREB and STAT5 control the activity of the promoter and/or CNS2 (refs 16, 18, 19). Furthermore, FOXO1 and FOXO3, two members of the FOXO TF family, bind to and activate the *foxp3* promoter and CNS2 (refs 20, 21, 22). Because FOXO proteins are inactivated by phosphorylation via a signalling axis formed by the molecules PI3K–Akt–mTOR^23^, enhanced binding of FOXO proteins to CNS2 explains upregulated FOXP3 expression upon interference with Akt/mTOR^24^,^25^,^26^. Very recent evidence demonstrated that the Tec family tyrosine kinase Itk influences mTOR signalling and that Itk-deficient animals have increased numbers of Tregs^27^. Apart from Akt/mTOR, the signalling molecules MEK/ERK and PKC-θ are also implicated in iTreg homoeostasis, as suggested by higher iTreg frequency when these pathways are inhibited^28^,^29^,^30^,^31^.

Therapy of autoimmune diseases is still challenging and requires novel strategies. The application of iTregs is considered as a new treatment option. However, iTregs can be unstable *in vivo*^32^,^33^,^34^ and even revert to cells which contribute to rather than suppress autoimmunity^33^,^34^, although such instability apparently depends on the disease model or experimental condition^35^. *In vivo* instability may be reflected by *in vitro* downregulation of FOXP3 in iTregs under conditions of continuous TCR stimulation but absence of TGFβ (refs 28, 32, 36). Recent evidence indicates that an ongoing TCR-signal transmits a negative signal for FOXP3 expression, because continuous culture without TCR-signal is sufficient to maintain FOXP3 expression^28^,^32^. In the present report, we characterize this negative feedback loop and decipher TCR-mediated dephosphorylation of STAT5 via the phosphatase PTPN2 along with downregulation of FOXO1 expression as its decisive components.

## Results

### A TCR-mediated suppressive pathway for FOXP3 expression

**Figure 1:**
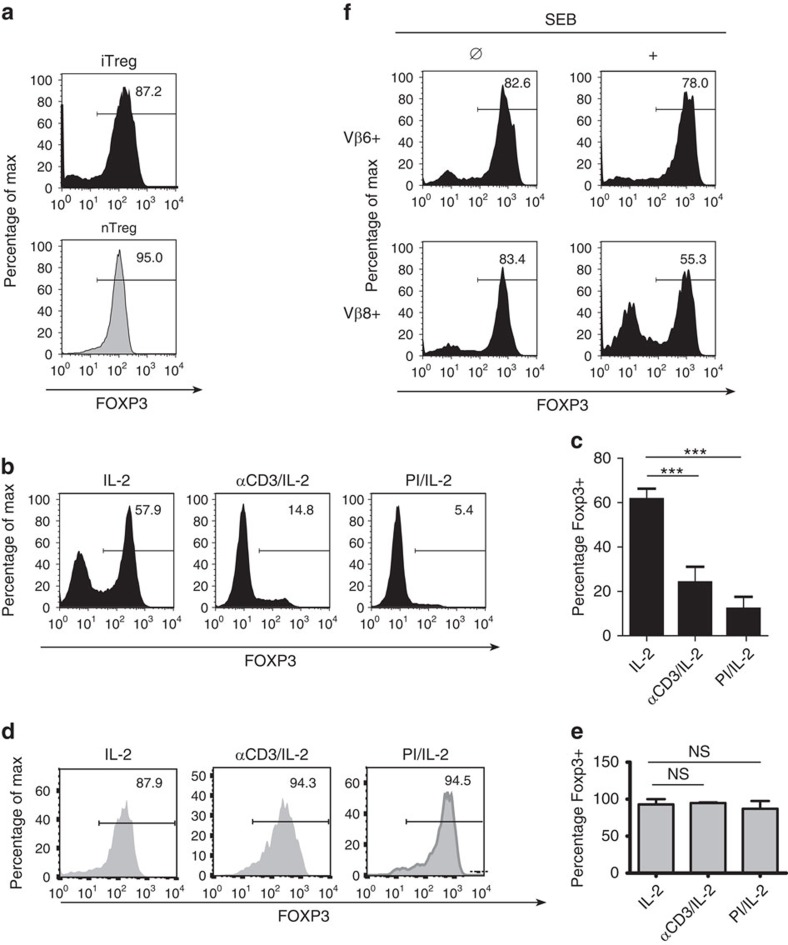
TCR-signalling suppresses FOXP3 expression in iTregs but not *ex vivo* purified Tregs. (**a**–**f**): intracellular staining of FOXP3; numbers indicate percentages of FOXP3^+^ cells according to the indicated range gate settings. (**a**) iTregs generated by 72 h stimulation via CD3/28 plus IL-2/TGFβ or GFP^+^ CD4^+^ nTegs sorted from DEREG mice, respectively. (**b**–**e**) re-stimulation of the cells depicted in (**a**) for 48 h by anti-CD3 or PMA/Ionomycin (PI), in the presence of IL-2. (**c**,**e**) Statistical evaluation plus s.d. (Student's *t*-test) of five (**b**,**c**) or three (**d**,**e**) consecutive experiments. ***P<0.001. (**f**) Ly 5.1+ iTregs re-cultured with IL-2 and Ly 5.1 negative congenic APC, with or without SEB, stained with anti-Ly5.1, anti-Vβ8 or anti-Vβ6 and gated for Ly5.1^+^ cells. Two experiments with similar outcome. ns: not significant.

We first confirmed reports by others^28^,^32^ that the TCR creates a dominant negative signal for maintenance of FOXP3 expression in iTregs but not in *ex vivo* prepared Tregs. In our study, these are mixtures of tTreg and pTreg and will be termed nTreg. As shown in Fig. 1a, high levels of FOXP3 were observed in nTregs, sorted as green fluorescence protein (GFP) positive cells from DEREG mice^37^. These mice contain a BAC transgene encoding the regulatory domains of *foxp3* upstream of *gfp*. Therefore, GFP positivity reflects active transcription of *foxp3*. FOXP3 was similarly expressed in iTregs induced from normal CD4^+^ wild-type (WT) cells after stimulation for 72 h via antibodies to CD3/CD28 (αCD3/28) in the presence of IL-2 and TGFβ. These antibodies recognize the CD3 complex of the TCR or the co-stimulatory molecule CD28, respectively, and can serve as mimic of antigenic recognition. After 72 h, iTregs were washed and further cultured in the presence of IL-2 and with or without re-stimulation via αCD3. Throughout this manuscript, we will refer to this culture period of iTreg as ‘re-culture'. As published before, re-culture with αCD3 for 48 h profoundly suppressed FOXP3 expression compared with re-culture without the TCR-signal (Fig. 1b,c). Importantly, CFSE staining confirmed that FOXP3 downregulation was regulated independently of cell proliferation, that is, was not an artefact caused by outgrowth of FOXP3 negative cells (Supplementary Fig. 1A). Downregulation of FOXP3 was also observed with PMA/Ionomycin instead of αCD3 to imitate intracellular signalling aspects of CD3 (Fig. 1b,c). In contrast to iTregs, neither αCD3 nor PMA/Ionomycin caused downregulation of FOXP3 expression in nTregs (Fig. 1d,e).

In theory, the above described finding could have been secondary to a soluble mediator like a cytokine induced by stimulation with αCD3/28. To rule this out, we re-cultured iTregs in the absence of αCD3 or PMA/Ionomycin, but presence of staphylococcal enterotoxin B (SEB) and antigen presenting cells (APCs). The superantigen SEB is recognized by all TCRs carrying a Vβ gene of the Vβ8 family, but not by Vβ6 family members. As shown in Fig. 1f, Vβ8+ and Vβ6^+^ iTregs kept high levels of FOXP3 after re-culture without SEB, while in its presence Vβ8^+^ but not Vβ6^+^ iTregs downregulated FOXP3 to a great extent. This demonstrates that the negative signal is also delivered by TCR-mediated recognition of an antigen presented by major histocompatibility complex molecules. Collectively, these data show that stimulation of the TCR generates an active negative signal for FOXP3 expression in iTregs, but not in nTregs.

To understand the underlying mechanisms, we next analysed molecules previously connected to FOXP3 regulation. Inhibitors of the signalling molecules PKC and MEK/ERK rescued FOXP3 expression during the re-culture period strongly or partly, respectively (Supplementary Fig. 2A). Although inhibition of PI3K by itself had no impact, it synergized with MEK/ERK for almost complete reversion of the negative TCR-signal. Thus, the previously reported negative effects of PKC, MEK/ERK and PI3K–Akt–mTOR on FOXP3 expression during generation of iTreg are likely embedded in the herein analysed TCR-initiated pathway. As anticipated, interference with proximal TCR-signalling by an inhibitor of the kinase Src also rescued FOXP3 expression. The pan inhibitor PS-1145 of the NFκB TF family, which also blocks c-Rel, had no effect (Supplementary Fig. 2B), while it reduced the luciferase activity in 293-mTLR9-luc cells. These cells report NFκB activity by enhanced expression of luciferase and were used as control for the activity of PS-1145 (Supplementary Fig. 2C). This result demonstrates that the suppressive TCR-activity acts independently of the published FOXP3-agonistic activity of c-Rel^11^,^12^,^13^,^14^. Similarly, cyclosporine A (CsA) did not interfere with FOXP3 downregulation (Supplementary Fig. 2A). Thus, the FOXP3-depleting TCR-activity acts independently not only of c-Rel but also of the phosphatase calcineurin and thus of NFAT TFs.

### The TCR signal interferes with active FOXP3 production

**Figure 2:**
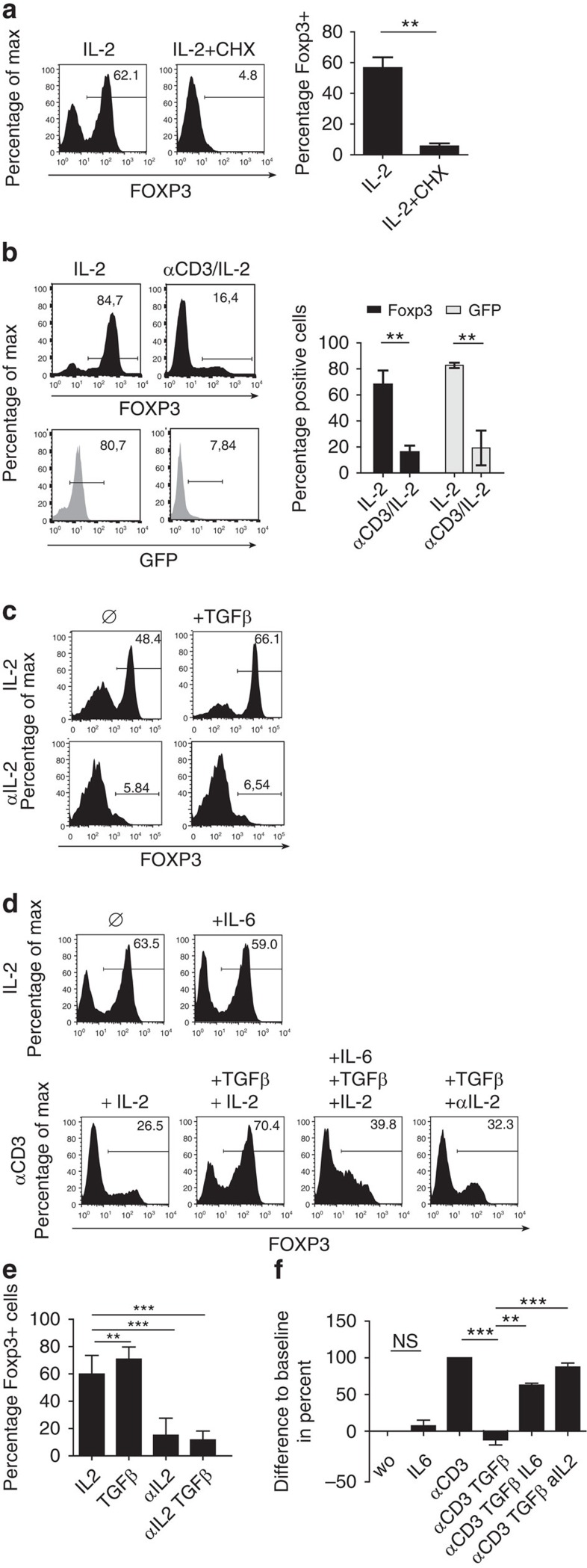
TCR-signalling interferes with active FOXP3 production. (**a**,**b** (top panels), **c**,**d**): intracellular staining of FOXP3 after 48 h of re-culture. (**a**) WT iTregs generated as before were re-cultured with IL-2, but without αCD3 and with or without CHX (10 μg ml^−1^). (**b**): iTregs were generated from sorted GFP-negative CD4^+^ DEREG cells. After 72 h, GFP^+^ cells were again sorted to a purity of >95% (range gate settings as depicted in **b**), and re-cultured for 48 h with IL-2 and with or without αCD3 (**b**, lower panels: GFP expression). (**a**,**b**) Statistical evaluation plus s.d. (Student's *t*-test) of three consecutive experiments. (**c**,**d**) WT iTregs were re-cultured for 48 h with IL-2, αIL-2, TGFβ or IL-6 with or without αCD3, as indicated. (**d**) Baseline represents cells re-cultured with IL-2 only. Five (**c**) or three (**d**) experiments with similar outcome. Statistical evaluation ± s.d. of (**c**) in (**e**) and of (**d**) in (**f**). n.s.: not significant. **P< 0.01; ***P< 0.001.

To analyse whether the TCR-signal led to instability of FOXP3 protein or interfered with *foxp3* transcription/translation, iTregs were re-cultured with or without cycloheximide. Presence of this protein synthesis inhibitor entirely blocked FOXP3 expression even in the absence of the TCR-signal (Fig. 2a). Thus, persistence of FOXP3 in iTregs relies on active *de novo* production, a process most likely blocked by the TCR-signal. To confirm this concept, we induced iTreg from sorted GFP-negative non-regulatory CD4^+^ T-cells of DEREG mice. After 72 h of induction of iTreg in these cells, GFP^+^ iTregs were sorted again and re-cultured with or without TCR-signal, as described above. As explained before, GFP positivity of these cells reflects active transcription of *foxp3*. Despite similar viability, most of these iTregs expressed GFP in the absence of the TCR-signal, but lost reporter gene expression after TCR stimulation (lower panels in Fig. 2b). FOXP3 protein expression was suppressed by the TCR signal as before (upper panels in Fig. 2b). Using a modified protocol with a 48 h resting period in the absence of the TCR signal between iTreg induction an re-culture, we confirmed these results for sorted RFP^+^ iTregs (Supplementary Fig. 3A) from FIR mice which encode the gene for RFP after an IRES sequence located in the endogenous *foxp3* locus. Because of the signal strength of RFP, downregulation of RFP could here only be determined by the mean fluorescence intensity (MFI). Using FIR cells, we also confirmed with RFP^+^ iTreg that FOXP3 downregulation occurs independently of cell proliferation (Supplementary Fig. 1B). Together, these results from both types of reporter cells clearly indicate suppressed gene transcription/translation as the reason for downregulation of FOXP3 protein. In addition, these findings exclude that lack of FOXP3 expression after stimulation with αCD3 is because of an hypothetical overgrowth of contaminating conventional non-regulatory CD4^+^ T-cells.

TGFβ and IL-2 are crucial for induction of *foxp3* gene transcription. To test their contribution for FOXP3 maintenance after the induction period and in the absence of the TCR-signal, we re-cultured WT iTregs without αCD3, but added either exogenous IL-2 and/or TGFβ or anti-(α)IL-2 antibodies. Although TGFβ further increased FOXP3 expression, αIL-2 totally blocked it (Fig. 2c,e). Notably, the FOXP3 enhancing effect of exogenous TGFβ was also neutralized by αIL-2. These findings demonstrate that *foxp3* maintenance can almost totally be traced back to mutually dependent activities of TGFβ and IL-2.

In a next step, we tested the influence of TGFβ and IL-2 on FOXP3 expression during TCR stimulation. We also analysed the effect of IL-6, which in combination with TGFβ induces pro-inflammatory Th17 cells and thus counteracts the *foxp3* inducing effect of TGFβ. As anticipated, TGFβ abolished the TCR-mediated block of *foxp3* transcription during re-culture (Fig. 2d,f). Again, this TGFβ activity depended almost totally on the presence of IL-2. IL-6 led to the known downregulation of FOXP3 even in the presence of TGFβ. Very interestingly however, IL-6 had almost no effect on the high FOXP3 levels observed after re-culture without TCR-signal. These results were confirmed with sorted RFP^+^ iTreg (Supplementary Fig. 3B). Clearly, IL-6 does not suppress FOXP3 expression by itself but rather interferes with the activity of TGFβ which abrogates TCR-mediated FOXP3-depletion. Because the amounts of FOXP3 seen in the absence of the TCR-signal are also dependent on TGFβ but not influenced by IL-6 (Fig. 2d,f), these findings suggest two qualitatively different TGFβ-mediated signals. Likely, binding of the TF Smad3 to CNS1 reflects the well known activity of TGFβ, which cooperates with STAT5 and is not influenced by IL-6. The novel TGFβ activity becomes only visible in the presence of the TCR-signal and can be counteracted by IL-6.

### The TCR-signal interferes with STAT5 phosphorylation

**Figure 3:**
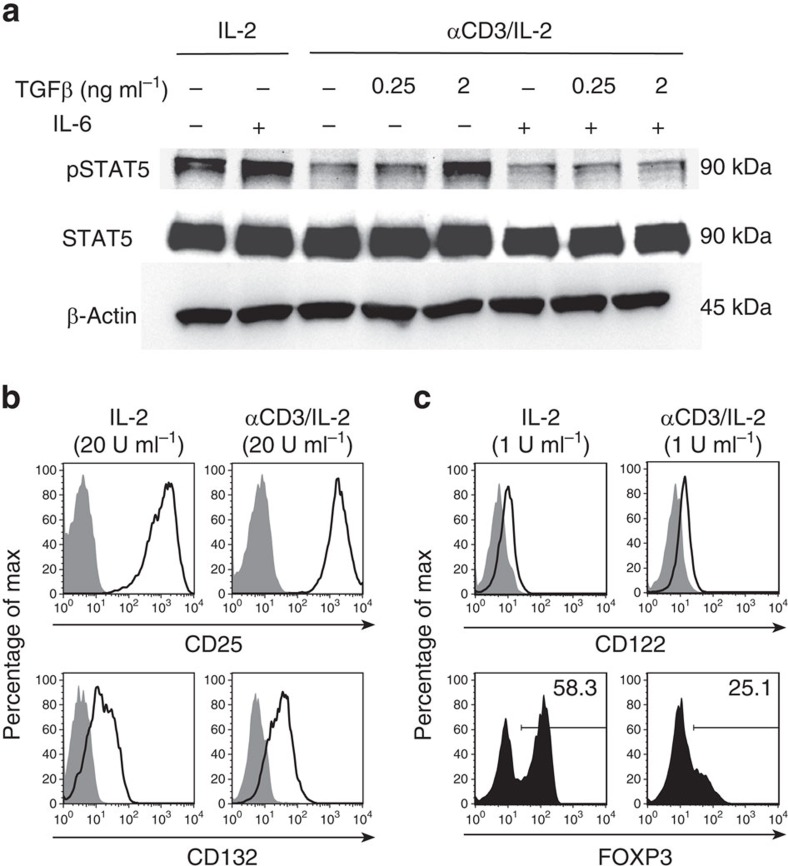
TCR stimulation hampers IL-2 signalling. (**a**) Western blot for pSTAT5, STAT5 and β-actin in lysates of iTregs re-cultured for 24 h under the indicated conditions, deprived of IL-2 for 1 h and resupplied with IL-2 (200 U ml^−1^) for 10 min. (**b**,**c**) Staining of surface CD25, CD122 or CD132 (open areas) vs isotype controls (grey areas) or of FOXP3 on iTreg re-cultured for 48 h in the presence of IL-2 (**b**: 20 U ml^−1^; **c**: 1 U ml^−1^) with or without αCD3. (**a**) Four, (**b**,**c**) two experiments with similar outcome.

Sixteen years ago, a report demonstrated that a TCR-trigger interfered with STAT5 signalling in human T-cells^38^. To test for such a mechanism in our setting, iTregs were re-cultured for 24 h in the presence or absence of the TCR-signal as before. After washing, the cells were deprived of IL-2 for 1 h, re-incubated for 10 min with IL-2 and the amounts of STAT5 phosphorylated on tyrosine residue Tyr694 (pSTAT5) were visualized by western Blot. Without TCR-signal, phosphorylation of STAT5 was induced by IL-2 (Supplementary Fig. 4A). Importantly, presence of the TCR-signal (Fig. 3a, Supplementary Fig. 4A) drastically blocked STAT5 phosphorylation. This result was confirmed by using sorted RFP^+^ iTreg (Supplementary Fig. 4B). Importantly, simultaneously added TGFβ dose-dependently maintained the potential to phosphorylate STAT5 (Fig. 3a). This finding illustrates that the above identified new biological activity of TGFβ can be characterized as licensing of STAT5 phosphorylation even in the presence of the TCR-signal. IL-6 in turn counteracted this licensing activity of TGFβ, resulting in the re-established block of STAT5 phosphorylation by TCR-signalling.

Inhibition of STAT5 phosphorylation could be secondary to downregulation of components of the IL-2 receptor (IL-2R). To test this, iTregs were re-cultured with or without TCR-signal as before and were analysed for expression of CD25, CD122 and CD132 by FACS analysis. We found no influence of the TCR-signal on expression of CD25 and CD132 (Fig. 3b). As for CD122, we faced the problem that IL-2 added during re-culture blocked antibody staining (Supplementary Fig. 5A). Therefore, we repeated the experiment in the presence of considerably lower amounts of IL-2. Under these conditions, FOXP3 levels were still quite high in the absence of the TCR-signal and completely downregulated in its presence (Fig. 3c, lower panels). With these limiting amounts of IL-2, staining with αCD122 was not influenced and no difference of CD122 expression in the presence or absence of the TCR-signal was found (Fig. 3c, upper panels). We confirmed this result by quantitative PCR with reverse transcription (qRT–PCR) analysis of CD122 mRNA after re-culture in the presence of the high concentrations of IL-2 used before (Supplementary Fig. 5B). Thus, inhibition of STAT5 phosphorylation cannot be explained by lack of components of the IL-2R.

**Figure 4:**
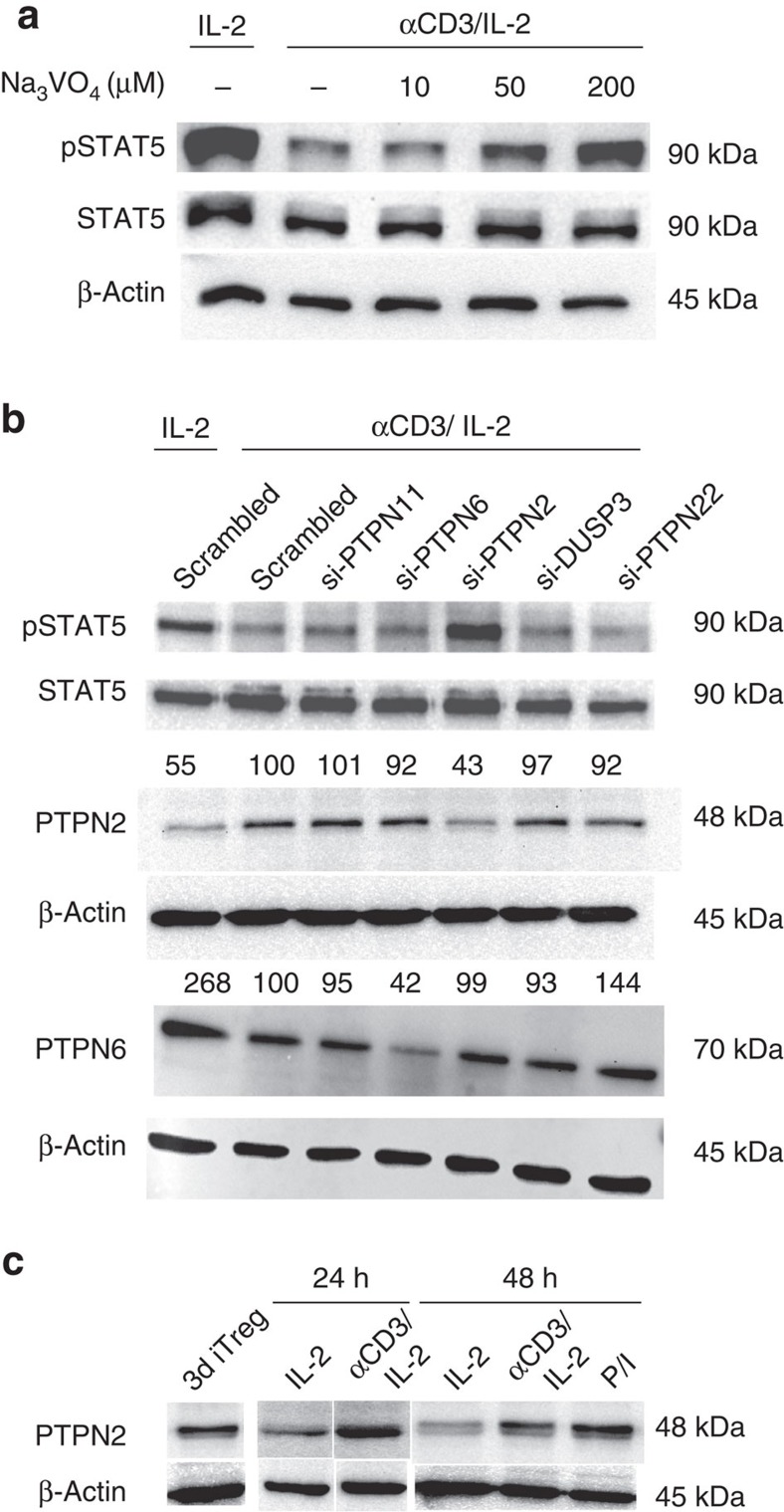
The p-Tyr phosphatase PTPN2 interferes with STAT5 phosphorylation. (**a**) Experiments similar as described in Fig. 3a were performed, however IL-2 was deprived for 90 min with application of the indicated concentrations of Na_3_VO_4_ for the last 45 min and resupply of IL-2 (200 U ml^−1^) for 10 min. Three experiments with similar outcome. (**b**) iTregs were induced as before, nucleofected with different siRNAs (one type per lane) directed against the indicated panel of p-Tyr phosphatases or with scrambled control siRNA and further incubated with IL-2 and without TCR-signal for 24 h, before being deprived of and resupplied with IL-2 as in A. Western blots were stained with antibodies to pSTAT5, total STAT5, PTPN2 or PTPN6. Numbers refer to relative values of densitometry. (**c**) western blot for PTPN2 in iTregs either immediately after 72 h induction (‘3D iTreg') or after 24 or 48 h re-culture with IL-2 and with or without αCD3. One representative of Figs 4b and 5c independent experiments.

Tyrosine phosphatases are obvious candidates to explain abrogated Tyr694 STAT5 phosphorylation during TCR-engagement. Indeed, several tyrosine phosphatases such as CD45, PTPN1 and PTPN2 have previously been linked with STAT5 (refs 39, 40, 41, 42). To investigate the involvement of a phosphatase, we deprived iTreg of IL-2 as before and subsequently treated them for 45 min with the tyrosine phosphatase inhibitor Na_3_VO_4_. Thereafter, IL-2 was added for 10 min and lysates were prepared for western blotting. Importantly, Na_3_VO_4_ dose-dependently restored the ability of IL-2 to phosphorylate Tyr694 of STAT5 to normal levels (Fig. 4a). These findings indicate that TCR-signalling induces the activity of a phosphatase that blocks STAT5 phosphorylation. Of note, the toxicity of the phosphatase inhibitor did not allow to directly measure its effect on FOXP3 expression during the long re-culture period.

To identify the phosphatase involved, we next transfected iTregs with a commercially available panel of siRNAs directed against different p-Tyr-phosphatases. To do this, iTregs were induced as before, removed from the TCR-signal, nucleofected with the siRNAs and 24 h later were re-stimulated via αCD3. Thereafter, cells were deprived of and resupplied with IL-2 as described above, and STAT5 phosphorylation was determined. As shown in Fig. 4b, siRNAs directed to PTPN1, PTPN6, PTPN22 and DUSP3 had no effect. In contrast, siRNA targeting PTPN2 entirely rescued pSTAT5 generation in response to IL-2 even after provision of the otherwise suppressive TCR-signal (Fig. 4b). The specificity of PTPN2 knockdown was confirmed with scrambled siRNA or the siRNAs against the other p-Tyr-phosphatases and verified by detection of PTPN6 instead of PTPN2 (Fig. 4b). PTPN2 knockdown was verified by western blot (Fig. 4b).

Given the involvement of PTPN2 in the FOXP3-depleting TCR-signal, we wondered if the TCR regulates the amounts of PTPN2 in iTregs. To address this question, iTegs were again re-cultured with or without the TCR-signal and cell lysates were tested for quantity of PTPN2 protein by Western blot. Remarkably, re-culture in the presence of αCD3 for 24 or 48 h upregulated PTPN2 protein compared with culture in IL-2 only (Fig. 4c). A similar activity was noted in the IL-2-deprivation experiment described before (Fig. 4b) and for PMA(Ionomycin (Fig. 4c). No upregulation of PTPN2 was found in nTreg after 48 h of *in vitro* stimulation (Supplementary Fig. 6A).

### The TCR-signal interferes with FOXO1 expression

In the context of clonal expansion of conventional T-cells, it has recently been described that the amount of FOXO1, a TF upregulating *foxp3* by binding to its promoter and to CNS2 (refs 20, 21, 22), is also downregulated by TCR-signals, a process involving microRNA miR-182 (ref. 43). In addition, FOXO TFs upregulate their own transcription, if they are not inhibited by phosphorylation, for example, in response to Akt^23^,^44^. Interestingly, TGFβ also interferes with Akt activity^45^, thus creating a potential scenario for yet another FOXP3 protecting activity of this cytokine. Finally, ERK was shown to phosphorylate FOXO1 independently of Akt^46^, a finding possibly explaining the previously described ERK activity during TCR-mediated downregulation of FOXP3 (ref. 28).

**Figure 5:**
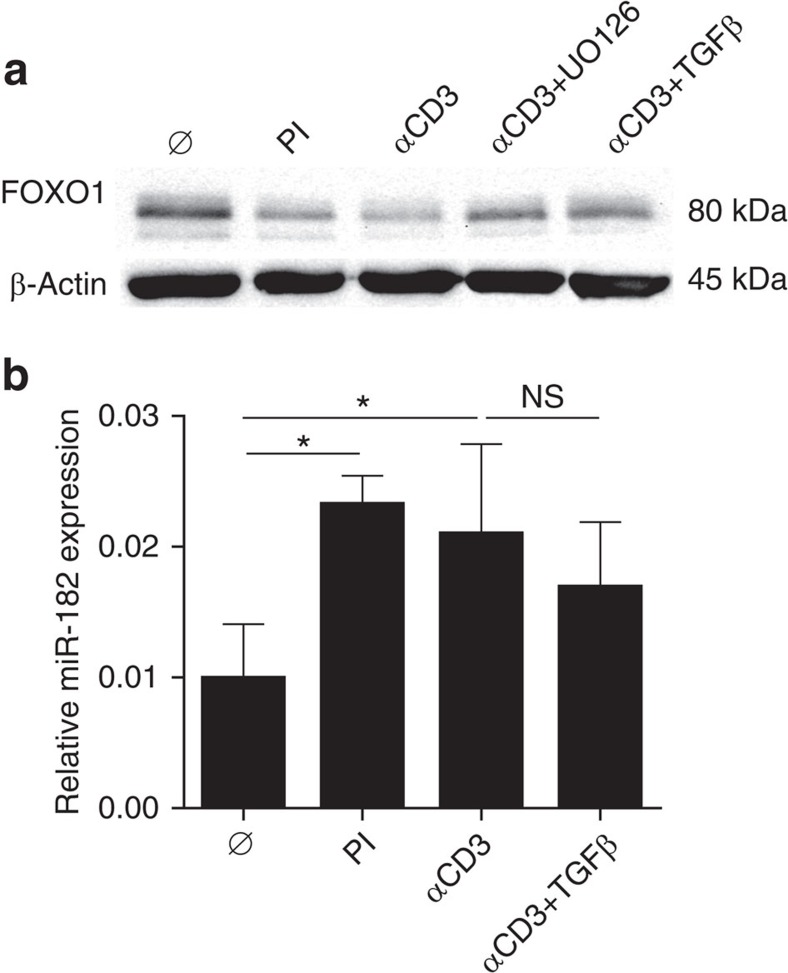
TCR stimulation upregulates miR-182 and suppresses FOXO1. (**a**) Western Blot for FOXO1 and β-actin in lysates of iTreg re-cultured for 24 h in the presence of IL-2 under the indicated conditions. Three experiments with similar outcome. (**b**) qRT–PCR analysis of miR-182 expression in iTreg re-cultured for 48 h with IL-2 under the indicated conditions. Statistical evaluation plus s.d. (Student's *t*-test) of four separate experiments. *P<0.05. ns: not significant.

To test for these options, the amount of FOXO1 protein was analysed in cell lysates of iTregs after 24 or 48 h of re-culture. As shown in Fig. 5a and Supplementary Fig. 6B, [Fig f6], the TCR-signal strongly downregulated FOXO1 protein. No alteration in FOXO1 expression was observed in nTreg (Supplementary Fig. 6A). The effect in iTreg was inhibited by TGFβ (Fig. 5a) and, to a similar extent, by inhibition of ERK via the MEK-inhibitor UO126. Accordingly, combined inhibition of ERK and Akt (via PI3K) synergistically protected FOXP3 expression in the presence of the TCR-signal (Supplementary Fig. 2A). Addition of PMA/ionomycin had a similar effect as stimulation with αCD3. At the same time, αCD3 and PMA/ionomycin upregulated the expression of miR-182 (Fig. 5b). This process was also slightly influenced by TGFβ, but without statistical significance. Thus, the TCR-signal interferes with FOXP3 induction not only by disturbing STAT5 phosphorylation but also by reducing the expression of FOXO1. Because miR-182 regulates FOXO1 (ref. 43), this effect of the TCR is partially caused by upregulation of miR-182 (Fig. 5b). In addition, it likely involves phosphorylation of FOXO1 via ERK and Akt to hinder binding and transcriptional activity of FOXO1 at its own *foxo1* promoter. The finding that TGFβ conserves FOXO1 quantities even in the presence of the TCR signal (Fig. 5a), represents a third activity of this cytokine to support expression of FOXP3.

### Active FOXO1 plus STAT5 restore FOXP3 expression

**Figure 6 f6:**
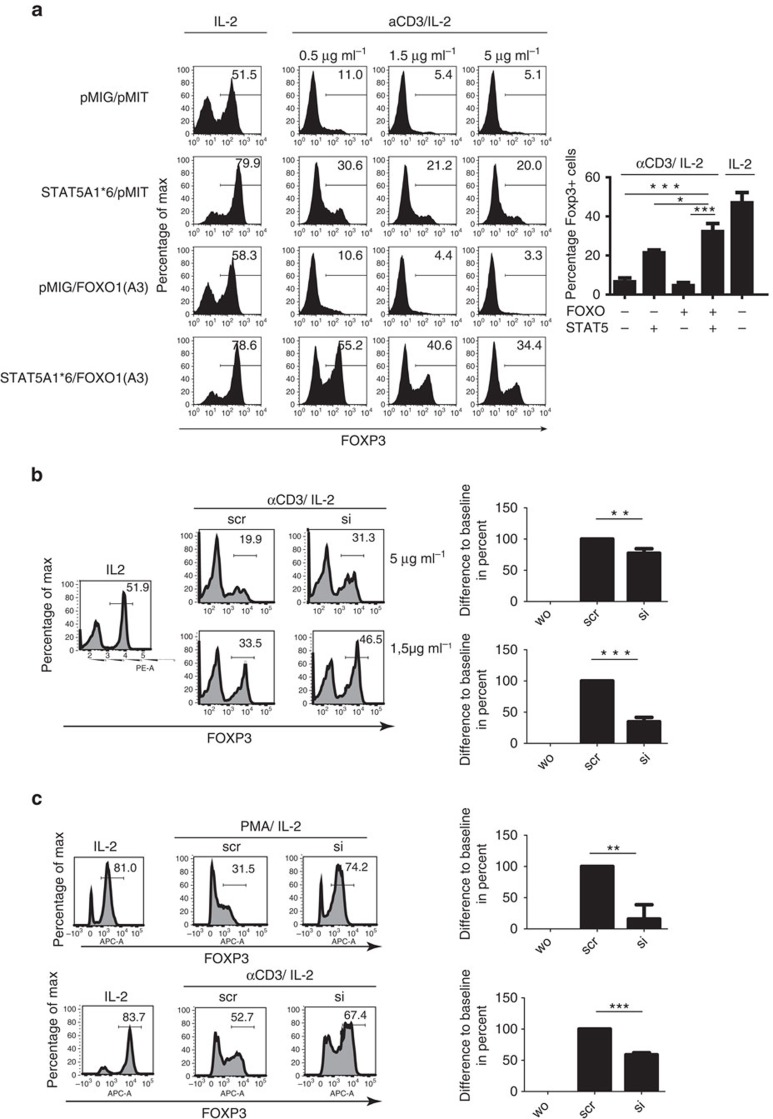
Overexpression of CA STAT5 and CA FOXO1 and knockdown of PTPN2 overcome the negative TCR-signal. (**a**) Primed iTregs were retrovirally transduced twice within 48 h with CA STAT5 or CA FOXO1 or the respective controls pMIG or pMIT, followed by 48 h of re-culture under the indicated conditions. Intracellular staining of FOXP3 in cells gated for successful double infection. Five experiments with similar outcome. Histograms from one representative experiment and summary (± s.d.) of the experiments with 5 μg ml^−1^ of αCD3. (**b**,**c**). Primed iTregs were nucleofected with siRNA against PTPN2 followed by further 24 h of culture without the TCR-signal and 24 h of re-culture under the indicated conditions (in (**c**): PMA:5 ng ml^−1^; αCD3: 0.5 μg ml^−1^). Intracellular staining of FOXP3. (**c**) RFP^+^ cells were sorted after the induction period (purity 98-99%). (**b**,**c**) The histograms are representative for three experiemts summarized in the respective diagrams (± s.d.) (Student's *t*-test) depicted to the right. Baseline represents cells re-cultured with IL-2 only. *P<0.05; **P<0.01; ***P<0.001.

We aimed to prove that STAT5 (via PTPN2) and FOXO1 were the decisive mediators of the TCR-induced FOXP3-depleting activity. In a first step, we tested if infection with retroviruses, which overexpress phosphorylation-independent and thus constitutively active (CA) forms of STAT5 or FOXO1, might rescue FOXP3 expression in the presence of the TCR-signal and absence of TGFβ. iTregs were generated as before and were infected with these retroviruses, either separately or combined, or with control viruses. Thereafter, cells were re-cultured, as before. Data in Fig. 6a demonstrate that, depending on the strength of the TCR signal (reflected by variable amounts of αCD3), only the combination of CA STAT5 and FOXO1 was able to restore FOXP3 expression to a great extent. In contrast, CA STAT5 on its own was less active, while FOXO1 alone had no detectable activity, similar as in cells that had been infected with control viruses only. This result was confirmed in sorted RFP^+^ FIR iTreg cells (Supplementary Fig. 7). The relative frequency of cells infected with none, one or both of the viruses also allowed us once again to confirm that downregulation of FOXP3 was not accompanied by outgrowth of a hypothetical FOXP3 negative cell population (Supplementary Fig. 8). Altogether, these data confirm that the above described pathways all merge to control FOXP3 expression by influencing either amount or phosphorylation of FOXO1 and STAT5, respectively.

In the next experimental setup, we replaced the retrovirus encoding CA STAT5 in the experimental setup described before by knocking-down PTPN2 via siRNA (Fig. 6b). iTregs were again re-cultured with or without the TCR-signal and lost FOXP3 expression in its presence. However, if cells were nucleofected with siRNA, FOXP3 expression was significantly restored even in the presence of the TCR-signal. These effects were observed at different concentrations of αCD3 and were again reproduced in sorted RFP^+^ FIR iTreg cells (Fig. 6c) and also, if αCD3 was replaced by PMA (Fig. 6c). As expected, FOXP3 recovery was not observed if scrambled control siRNA was used.

**Figure 7:**
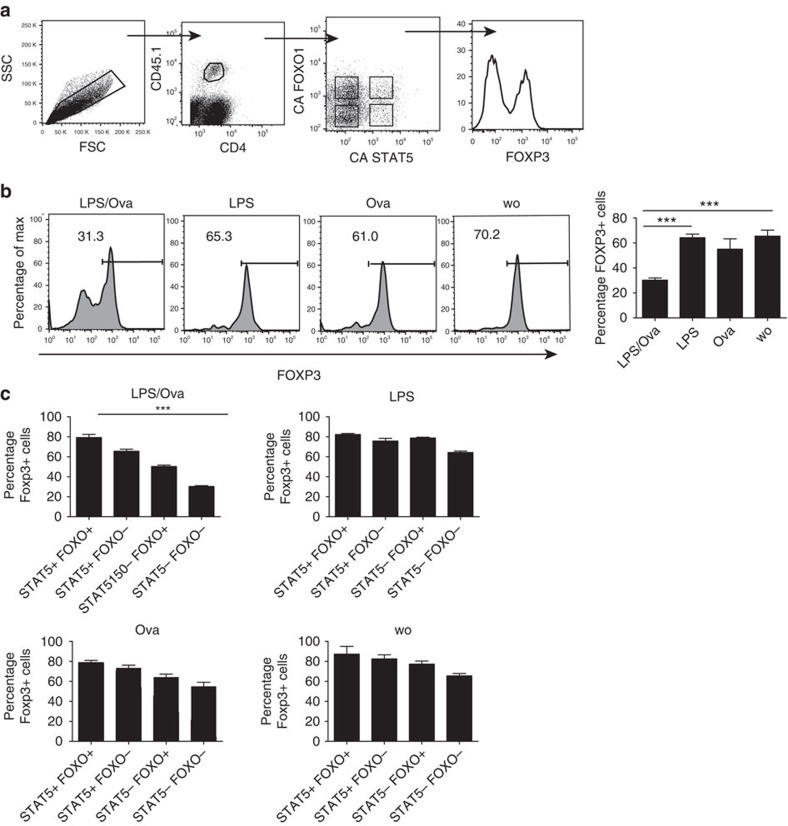
Antigen-specific downregulation of FOXP3 in iTregs *in vivo*. iTregs were induced from OT II/FIR cells, were transfected with either CA FOXO1 or CA STAT5 or both, sorted for RFP^+^ cells (purity 96%) and were transferred i.p into C57BL/6 recipient mice with or without OVA and/or LPS (four mice per group). Mesenteric LN cells were analysed 3 days later by flow cytometry. (**a**): Gating strategy. (**b**) FOXP3 staining of cells within the lower left (‘double negative') gate defined in the third panel in (**a**). Columns depict the mean ± s.d. of FOXP3^+^ (%) double negative cells of the different groups of mice. (**c**) Mean ± s.d. (Student‘s t-test) of FOXP3^+^ cells (%) transfected with CA STAT5 or CA FOXO1 or both of the different groups of mice. Two experiments with similar outcomes; the *P*-values analogous to those shown here were <0.01 in the second experiment.

Finally, we wanted to prove that the herein demonstrated TCR-mediated pathway is of relevance not only *in vitro*, but also *in vivo*. To do this, we generated iTregs from OT II T-cells which express a transgenic TCR reactive with ovalbumin (OVA). These cells which also expressed CD45.1 as a traceable congenic marker, were transferred into CD45.2 expressing recipient mice. These iTregs had been infected *in vitro* with the above described viruses overexpressing CA STAT5 or CA FOXO1. After transfer, the mice were injected i.p.with OVA and or LPS as adjuvant. After 3 days, cells from mesenteric lymph nodes of these mice were stained for CD4, CD45.1, FOXP3 and for the markers confirming the respective retroviral transduction. The gating strategy is depicted in Fig. 7a.

When analysing the non-infected (‘double negative') cells in all groups of mice, we found that the combination of LPS and OVA led to strong downregulation of FOXP3. In contrast, mice injected with LPS or OVA separately, only showed small reductions compared with mice without treatment (Fig. 7b). Furthermore, we noted a strong cell expansion in the presence of LPS, with or without OVA, while only low numbers of transferred cells were detectable in mice without LPS. Their low frequency precluded the acquisition of enough cells simultaneously infected by both viruses (‘few events', f.e.), while there were still enough cells infected with either one of the viruses separately. With this limitation, it became apparent (Fig. 7c) that CA STAT5 was able to restore FOXP3 expression to some extent. CA FOXO1 had a small but significant effect only in LPS/OVA-injected mice. Co-expression of CA STAT5 and CA FOXO1 totally restored FOXP3 expression in mice injected with LPS/OVA.

Because of the strong expansion of cells after LPS application, we wanted to confirm again that our findings were not explained by outgrowth of FOXP3 negative cells. We therefore crossed FIR and OT II mice. RFP^+^ FIR/OT II iTreg of these mice were induced, sorted and the above described experiment was repeated; however, the mouse group receiving only OVA was omitted because of limited cell numbers. The main findings of the previous *in vivo* experiment were all confirmed (Supplementary Fig. 9). Strong downregulation of FOXP3 was deteced after application of LPS/OVA, while LPS alone had no effect, compared with mice receiving neither LPS nor OVA. Again, this downregulation was partly eliminated by separate overexpression of CA STAT5 or CA FOXO1, but fully neutralized by their combined overexpression. Together, these experiments prove that the herein described TCR-initiated mechanism responsible for FOXP3 downregulation, also operates *in vivo* and is not an *in vitro* artefact.

## Discussion

Despite considerable progress in recent years, treatment of autoimmune diseases remains a challenging task, regardless whether they occur spontaneously or are a collateral damage of treating another disorder, such as during graft-versus-host disease. iTregs are an attractive novel therapeutic concept for therapy-resistant autoimmune diseases, because they can be induced *in vitro* from peripheral blood T-cells, react with a wide range of different antigens and potently suppress effector T-cells. However, the stability of iTregs has been a matter of debate, especially after *in vivo* transfer. Several reports have demonstrated that in an inflammatory environment, iTregs may lose FOXP3 expression and turn into harmful inflammatory cells^33^,^34^.

In this report, we provide evidence that in conventional CD4^+^ T-cells, FOXP3 expression is under permanent stringent control of the TCR. As also reported by others^28^,^32^ and to our own surprise, the TCR-signal was not necessary for perpetuation of FOXP3 expression, but actually suppressed it continuously. Thus, the conditions used to generate iTregs on purpose *in vitro*, include at the same time signals for reversion of their phenotype. Previous reports studying mainly induction and not stability of iTreg *in vitro*, have demonstrated a FOXP3-suppressive signal mediated via the PI3K–Akt–mTOR axis^24^,^25^,^26^. However, when FOXP3 is not yet expressed, missing positive and active negative signals are much more difficult to be discerned from each other. Our experimental setup separates induction and test period and allows us to characterize the nature and potency of the suppressive TCR-signal.

Under such experimental conditions, our data show that the known role of TGFβ to upregulate *foxp3* transcription via the TFs Smad2/3 (ref. 17), is only one part of its activity. Rather, TGFβ has the additional role of neutralizing the suppressive TCR-signal, as also found by others^28^. This mechanism serves as a safe-guard to assure that iTreg activity is enhanced only in TGFβ-rich conditions which aim at immunosuppression. In contrast, during inflammation TGFβ cooperates with the pro-inflammatory cytokine IL-6 to generate inflammatory Th17 rather than iTreg cells, and FOXP3 is depleted. Remarkably, the TCR-counteractive activity of TGFβ is in itself a target of IL-6. Thus, IL-6 reduces FOXP3 expression not as much by an intrinsic transcriptional activity, but rather by interfering with the interplay of suppressive TCR-signal and its neutralization by TGFβ. Of course, the interplay between TCR, TGFβ and IL-6 is of high relevance for any therapeutic application of iTregs, because IL-6 is primarily present exactly under conditions, in which a therapeutic application of iTregs is attempted, namely during uncontrolled inflammation. Therefore, adoptive transfer of *in vitro*-induced iTregs may turn out to be more harmful than beneficial. Thus, it is of prime relevance to better understand the nature of the suppressive TCR-signal in order to potentially influence it for stabilisation of FOXP3.

In this report, we show that two TCR-triggered and separate pathways, which influence the mediators FOXO1 and STAT5, respectively, cooperate to suppress FOXP3 expression in response to the TCR-signal. The first pathway leads to downregulation of the TF FOXO1, which is known to bind to the *foxp3* promoter as well as to CNS2 and to act as an important inducer of *foxp3* transcription^20^,^21^,^22^. Certainly, the decrease in FOXO1 is particularly prominent due to the self-enhancing activity of FOXO1 for its own transcription^44^. Furthermore, the TCR-signal raises the presence of miR-182, which as we showed previously, blocks *foxo1* transcription^43^. The activity of FOXO1 is inhibited by phosphorylation via Akt^23^, which explains the above mentioned FOXP3-suppressive activity of the PI3K–Akt–mTOR pathway. Independently of Akt, FOXO1 is also phosphorylated by ERK^46^. These previous data fit to our results that combined inhibition of ERK and the PI3K–Akt–mTOR axis partially overcomes the negative TCR-signal for FOXP3 expression. We further show that one of the TCR-neutralizing activities of TGFβ is to raise expression of FOXO1, probably via its published ability to interfere with the Akt signal^45^.

**Figure 8:**
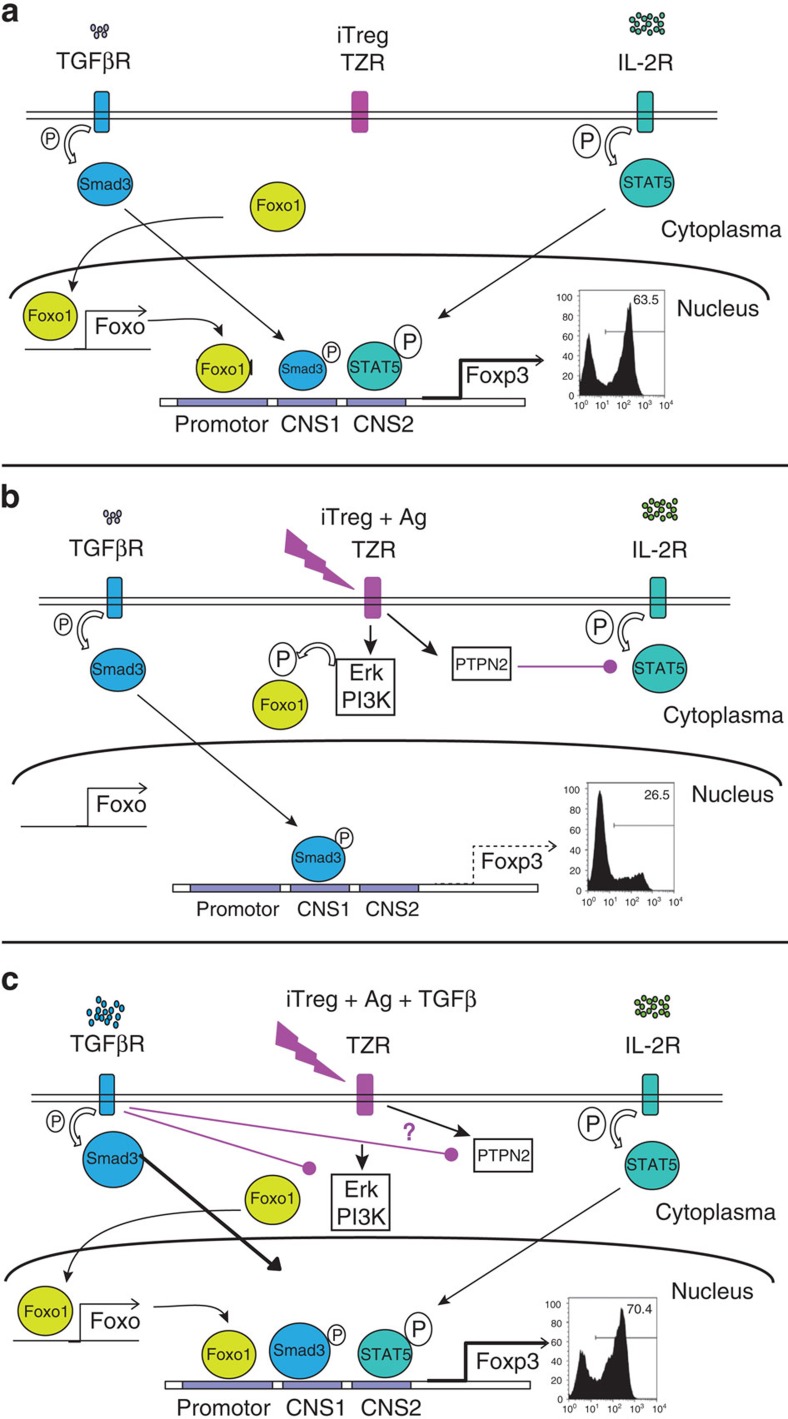
Cartoon summarizing the main findings. .

In addition to these activities on FOXO1, we find that the TCR-signal also interferes with Tyr-phosphorylation of the TF STAT5, which is essential for *foxp3* transcription^18^,^19^. This interference is not caused by a missing kinase, but rather by overexpression of the phosphatase PTPN2 in response to TCR-signalling. Accordingly, a blocker of p-Tyr phosphatases or specific knockdown of PTPN2 but not of other phosphatases re-established STAT5 phosphorylation despite presence of the TCR-signal. The decisive roles of STAT5 and FOXO1 during TCR-mediated FOXP3 depletion were confirmed by overexpressing CA and phosphorylation-independent versions of STAT5 and FOXO1, which were able to fully rescue FOXP3 expression even in the presence of the TCR-signal and absence of TGFβ. We further substantiated the crucial impact of PTPN2 in this process, because by depletion of PTPN2 via knockdown, FOXP3 expression was also partially restored. A cartoon summarizing our main findings on iTreg is shown in Fig. 8. In contrast to iTreg, nTreg showed unaltered expression of FOXO1 and PTPN2 in response to TCR-triggering, thus potentially explaining, why nTreg are resistant to TCR-mediated FOXP3 suppression.

Our data show that the mechanism used by the TCR to suppress FOXP3 expression includes elevations of miR-182 which downregulates *foxo1* and of PTPN2 which dephosphorylates STAT5. The need for tight regulation of this phosphatase is demonstrated by the early lethality of PTPN2-deficient mice^47^. In accordance with our results, mice with T-cell-specific PTPN2 deficiency harbour increased numbers of Tregs^48^, although the mechanism analysed by us was not addressed in that study. Despite slightly increased Treg frequencies, these mice suffer from an autoimmune syndrome^48^, demonstrating that PTPN2 also controls conventional T-cells. In this regard, PTPN2 limits lymphopenia-induced proliferation in conventional T-cells^49^. Of note, another study found reduced frequencies of Treg cells upon T-cell-specific deletion of PTPN2 (ref. 50). The difference between the two studies was explained^50^ by the time frame during which the promoters used (CD4 vs Lck) are active. In contrast to our work, both studies relate to the primary induction of Tregs, not to their regulation by PTPN2 once they are differentiated. In further confirmation of the importance of PTPN2, single-nucleotide polymorphisms in the human *PTPN2* gene are a risk factor for developing autoimmunity, although in that study^51^, reduced PTPN2 levels were for unknown reasons associated with reduced rather than enhanced STAT5 signalling.

In addition to mechanistic insights, our data explain, why iTregs may revert to pathogenic effector cells *in vivo.* Accordingly, we have demonstrated also *in vivo* that the antigen recognized by iTregs leads to downregulation of FOXP3 and that again, this effect can be neutralized by a combination of CA FOXO1 and STAT5. As a consequence, treatment of autoimmune diseases with *in vitro* derived iTregs may not be successful because of limited availability of TGFβ at the target organ or to dominant amounts of inflammatory IL-6. To maintain iTreg activity, it may be necessary to systemically apply TGFβ or other FOXP3 stabilizing components such as retinoic acid^52^. Alternatively, PTPN2 inhibitors or FOXO1-inducing compounds may be included during the induction phase *in vitro* in order to stabilize FOXP3 expression after *in vivo* transfer.

## Methods

### Mice

C57BL/6 were obtained from Harlan. DEREG mice^37^ and FIR (for FOXP3-IRES-mRFP)^53^ mice on the C57BL/6 background were provided by Tim Sparwasser (Hannover, Germany) and Karsten Kretschmer (Dresden, Germany). These mice as well as CD45.1-congenic OT II mice^54^ (expressing a transgenic TCR reactive with ovalbumin, OVA) were maintained in specific pathogen-free conditions in the BMFZ Marburg and female mice were used between 2 and 5 months of age. Animal experiments were approved by the Lower Saxony Committee on the Ethics of Animal Experiments.

### Cell preparation and *in vitro* stimulation

CD4^+^ T-cells were purified by magnetic cell sorting from spleens and lymph nodes and were primed with anti (α)-CD3 (5 μg ml^−1^) for 72 h as described^55^, in Click's RPMI medium (Biochrom) in the presence of αCD28 (clone 37.51; 0.5 μg ml^−1^), recombinant (r) human (h) IL-2 (Proleukin, 100 U ml^−1^) and rh TGFβ (Peprotech; 2 ng ml^−1^), αIL-4 (10% of culture supernatant (SN) of 11B11 cells) and αIFNγ (5 μg ml^−1^, purified from SN of XMG1.2 cells). After priming, cells were removed from the stimulus, washed and re-cultured for 24 or 48 h in 48 well (3 × 10^5^ in 0.5 ml medium) or 12 well (3 × 10^6^ in 2 ml medium) culture plates (Greiner) with or without pre-coated αCD3 (5 μg ml^−1^) in the presence of IL-2 and/or TGFβ and IL-6 (Peprotech; 10 ng ml^−1^). In some experiments, re-stimulation occurred in the presence of α-mouse IL-2 (clone S4B6; 40 μg ml^−1^) instead of IL-2 or of PMA (Sigma; 10 or 5 ng ml^−1^) plus Ionomycin (Sigma; 370 ng ml^−1^) instead of αCD3. Where indicated, cells were re-stimulated in the presence of PP2 (Alexis, 10 μM), cycloheximide (CHX, Sigma, 10 μg ml^−1^), UO126 (Calbiochem, 10 μM), AEB071 (Novartis, 3 μM), Ly294002 (Enzo, 10 μM), Cyclosporine A (CsA; Calbiochem, 10 ng ml^−1^), PS-1145 (Sigma, 10 μM) or SB-431542 (Biovision, 10 μM) after a 30 min preincubation with the respective reagent. CD4^+^GFP^+^ cells from DEREG mice or CD4^+^RFP^+^ cells from FIR mice comprise a mixture of tTreg and pTreg and are termed nTreg throughout this study. These cells were sorted via a FACSAria cell sorter (BD) and directly submitted to the re-stimulation protocol. In some experiments, CD4^+^GFP^−^ cells from DEREG mice were sorted via FACSAria and differentiated unter iTreg conditions for 3 days. Subsequently, GFP^+^ iTreg were again sorted and re-stimulated as indicated. To induce iTreg from FIR mice, CD4^+^ cells were purified by MACS and initially were further sorted for RFP negativity. Since these cells yielded similar results as MACS sorted cells still containing low frequencies of RFP^+^ nTregs, and because *ex vivo* sorted RFP^+^ nTreg showed no proliferation *in vitro* (in contrast to iTreg) we later omitted this sorting step. In all situations, RFP^+^ iTreg were induced for 72 h from FIR CD4^+^ T-cells, rested for 48 h in the absence of αCD3, sorted for RFP positivity and re-cultured as described before. To prepare the cells for pSTAT5 analysis by western blotting, they were re-stimulated for 24 h, washed and kept for 1 h in serum-free medium containing all stimuli and reagents used during these 24 h except for IL-2. Thereafter, IL-2 was added (200 U ml^−1^) for 10 min and lysates were prepared. In some experiments, the phosphatase inhibitor sodium vanadate (Na_3_VO_4_; Sigma, 10–200 μM) was added in an extra step for 45 min before addition of IL-2. In contrast, lysates for FOXO1 analysis were prepared directly after 24 or 48 h of re-stimulation. In some experiments, congenic Ly5.1^+^ iTregs (2 × 10^4^) were re-stimulated for 48 h in 96-well round-bottom culture plates (Costar) by SEB (Sigma, 10 μg ml^−1^) in 200 μl medium in the presence of Ly5.1^−^Ly5.2^+^ spleen cells (3 × 10^5^).

### Western blotting

Western blotting was performed as described^56^, however including the phosphatase inhibitors sodium fluoride (NaF; Merck, 20 mM) and Na_3_VO_4_ (200 μM) and antibodies against pSTAT5 (C11C5, Cell Signaling, 1:200), total STAT5 (C-17, Santa Cruz, 1 μg ml^−1^), total FOXO1 (C29H4; Cell Signaling, 1:1,000), β-actin (A5441;Sigma, 1:10,000) and PTPN2 (252294; R&D, 0.5 μg ml^−1^). Secondary reagents were goat-anti-mouse-HRP (sc-2055) and goat-anti-rabbit-HRP (sc-2004), each diluted 1:1,000, both from Santa-Cruz.

### qRT–PCR and flow-cytometric analysis

qRT–PCR to test for the quantities of miR-182 and FOXO1- mRNA was performed as described^43^. The following custom primers were used for SYBR Green-based real-time PCR (Roche): mouse Foxo1 forward, 5′-CGGGCTGGAAGAATTCAATTC-3′, and reverse, 5′-AGTTCCTTCATTCTGCACTCGAA-3′; mouse HPRT forward, 5′-TCCTCCTCAGACCGCTTTT-3′, and reverse, 5′-CATAACCTGGTTCATCATCGC-3′; and human HPRT forward, 5′-ACCCTTTCCAAATCCTCAGC-3′, and reverse, 5′-GTTATGGCGACCCGCAG-3′. miR-182 was amplified with the following primers: pri-182 forward, 5′-GTTAACGTTAACTGTGGGAAGAGCGC-3′ and reverse, 5′-CTCGAGAAAAAACACCGAGAAGAGGTCGA-3′. Expression values were normalized to hypoxanthine guanine phosphoribosyl transferase by the 2^−ΔCT^ method.

For flow-cytometric detection of intracellular FOXP3, cells were fixed with FOXP3 Fixation/Permeabilization reagent (eBioscience) for 20 min at 4 °C followed by intracellular staining with αFOXP3-PE (eBioscience) as published^56^. The three chains of the IL-2R were extracellularly stained using the antibodies Tm-b1-FITC against CD122 (5 μg ml^−1^), TUGm2-Biotin against CD132 (1 μg ml^−1^) and PC61.5-APC against CD25 (50 ng ml^−1^) (all by eBioscience). Thereafter, biotinylated αCD132 was visualized using streptavidin-PE (eBioscience, 660 ng ml^−1^). Cells re-stimulated by SEB (see above) were first incubated for 5 min with the αCD16/32 FcR blocking antibody (2.4G2; BD, 5 μg ml^−1^), followed by extracellular staining using either αVβ6 (50% of SN of 44-22-1 cells) plus α-rat-IgG-FITC or αVβ8 (F23.1; ascites, 1:100) plus α-mouse-IgG-Alexa488 (Life Technologies, 1:1,000), or the respective isotype controls. Thereafter, mouse IgG was added to block remaining binding sites of α-mouse-IgG-Alexa488, after which αLy5.1-Biotin was added and visualized by subsequent incubation with Streptavidin-PerCP. Finally, the cells were stained intracellularly for FOXP3. After retroviral infection (see below), the intracellular staining protocol was altered to avoid leakage of green fluorescent protein (GFP). First, the cells were stained extracellularly with αThy1.1-APC (HIS51; eBioscience, 1 μg ml^−1^) to detect infection with FOXO(A3) or its control virus. After washing, cells were fixed with 0,5% paraformaldehyde for 5 min at room temperature, followed by fixation with FOXP3 Fixation/Permeabilization reagent for 20 min at 4 °C and intracellular FOXP3 staining as described above. In some experiments, cells were labelled with CFSE (Sigma; 5 μM for 8 min at 37 °C) and after 24 h of re-culture were co-stained for FOXP3 as just described. Transduction with STAT5A1*6 or control virus was verified based on GFP expression. Cells prepared from mice after transfer of OT II iTregs (see below) were also stained with αCD45.1-PacBlue and αCD4-PerCP (both Biolegend, 1:300 and 1:400). Flow cytometry was performed on an Aria 3 machine (BD) using the FACS-Diva 6.1 software.

### siRNA treatment and retroviral transduction

Nucleofection of iTregs with siRNA was performed after the 72 h induction period and one further day of culture without αCD3, according to a published protocol^57^ using either commercially available siRNA directed against a panel of p-Tyr-phosphatases (L-040061-00-0005, L-041173-00-0005, L-041172-00-0005, L-047024-00-0005, L-055896-00-0005; ThermoFisher Scientific) or a mix of self-designed siRNAs against PTPN2 or scrambled control siRNA from IBA. The self-designed sequences were: PTPN2 #1: sense: 5′- oAoAUGUGCACAGUACUGGdCdCdAoAoCoG-3′; antisense: 3′- oUoUACACGUGUCAUGACCGGUdT-5′. PTPN2 #2: sense: 5′-oAoACUCAGAUUCUCCUACdAdTdGoGoCoC-3′; antisense: 3′-oUoUGAGUCUAAGAGGAUGUACdC-5′, where o stands for 2′-Methoxy and d for 2′Deoxy. Nucleofected cells were cultured for 24 h without TCR-signal in the presence of IL-2, and were then re-stimulated via αCD3 or PMA for 24 h. Therafter, FOXP3 expression was determined or the cells were deprived of and resupplied with IL-2 as described above. In the case of FIR cells, the same protocol was used; however, RFP^+^ cells were sorted after the induction period. As for retroviral transduction, the plasmid pMIT-FOXO1(A3) was provided by David Fruman, (Irvine, USA). For constitutive active STAT5A1*6, EcoRI was used to excise its sequence from pMSCV-STAT5A1*6-NGFR^58^ and clone it into the multiple cloning site of the retroviral pMIG-RI vector. After 72 h of priming, iTreg were removed from the TCR-signal and transduced with either pMIT-FOXO1(A3) or pMIG-STAT5A1*6 or the respective control vectors as described^56^, followed by a 24 h culture period with IL-2 (20 U ml^−1^), αIFNγ (5 μg ml^−1^) and αIL-4 (10% SN). The following day, this procedure was repeated. Subsequently, the cells were re-cultured for 48 h with or without αCD3, as described above. When using FIR T-cells, iTreg were induced as before, but retroviral transduction was performed during the final 24 h of the induction period and the first 24 h of the resting period and RFP^+^ cells were sorted immediately before re-culture.

### *In vivo* studies

iTreg were induced from CD45.1^+^ OT II or CD45.2^+^ OT II mice crossed with FIR mice (OT II/FIR) as before. These cells were transfected with the above described retroviruses on days 2 and 3 of the induction period. After resting for 3 days, cells were harvested and in the case of OT II/FIR cells were sorted for RFP positivity. Cells were then transferred intraperitoneally (i.p.) into recipient C57BL/6 mice (3 × 10^6^ cells per mouse) with or without OVA (100 μg per mouse) and LPS (30 μg per mouse). After 3 or 7 days, the mice were killed and mesenteric lymph node (LN) or spleen cells were analysed by flow cytometry.

### Luciferase assay

293-mTLR9-luc cells were cultured in 96-well microtiter plates in 100 μl medium with or without 2 μM CpG for 24 h in absence or presence of different concentrations of PS-1145, as published^57^. The next day, the cells were lysed in 50 μl (Dual-Luciferase ReporterAssay System, Promega) and an equal volume of substrate solution was added for luciferase measurement.

### Statistical analysis

Data were obtained from at least three independent experiments (mean±s.d.) and analysed with GraphPad Prism Version 5 software using Student's *t*-test (two-tailed) with *P*<0.05 considered as significant (*P<0.05; **P<0.01; ***P<0.001).

## Additional information

**How to cite this article:** Bothur, E. *et al*. Antigen receptor mediated depletion of FOXP3 in induced regulatory T lymphocytes via PTPN2 and FOXO1. *Nat. Commun.* 6:8576 doi: 10.1038/ncomms9576 (2015).

## Supplementary Material

Supplementary Information Supplementary Figures 1-12
