# Supplementary material for: Antigen receptor-mediated depletion of FOXP3 in induced regulatory T-lymphocytes via PTPN2 and FOXO1
Source: Nat Commun. 2015 Oct 13;6:8576. doi: 10.1038/ncomms9576 (PMC4633965; doi:10.1038/ncomms9576)

Supplementary Fig. 1

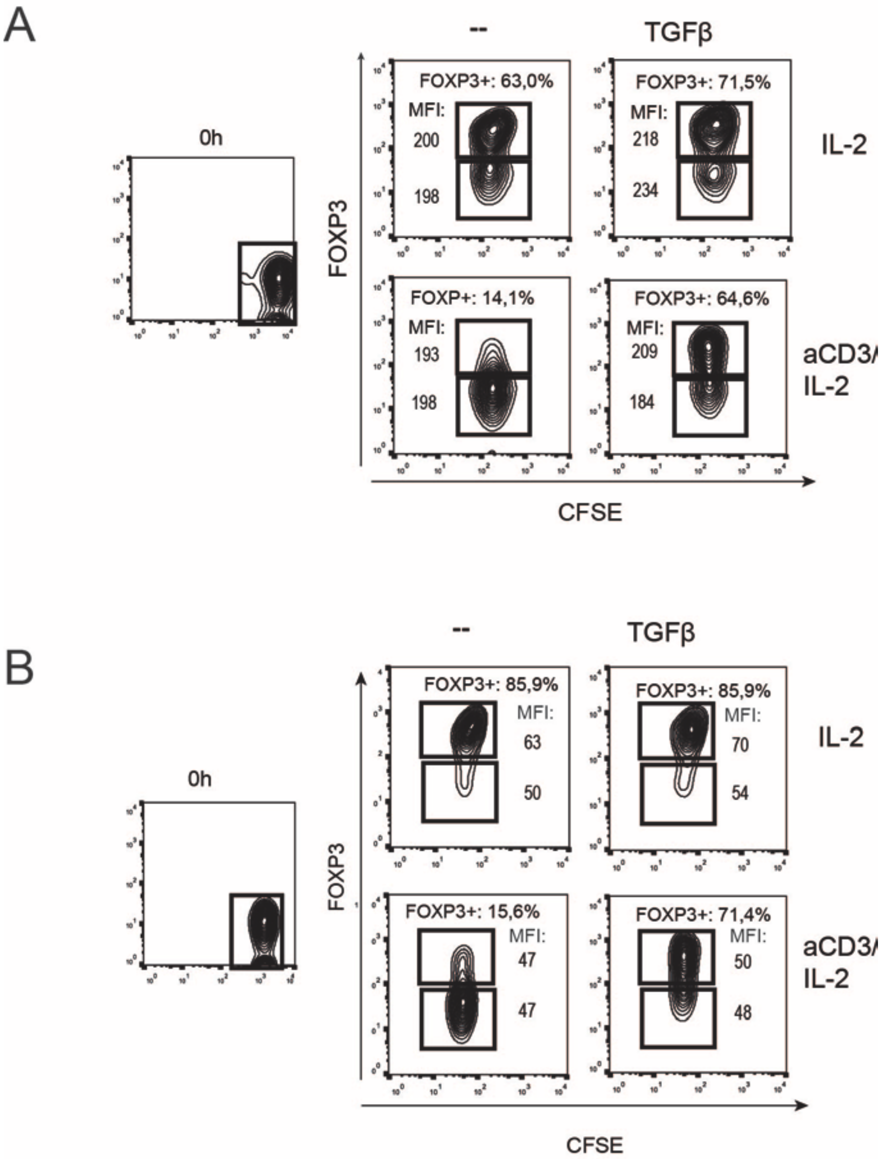

Supplementary Figure S1: **No relative growth advantage of Foxp3 negative cells.** iTreg were induced from WT (A) or FIR (B) $CD4^+$ T cells. FIR iTregs were then removed from the TCR signal for 48 h and RFP^+ cells were sorted (97%). Immediately after induction (A) or after sorting (B), the cells were labelled with CFSE and recultured in the indicated conditions for 24 h. Thereafter, Foxp3 was co-stained and the cells were analysed by flow cytometry. Two experiments for A and B, respectively, with similar outcome.

Supplementary Fig. 2

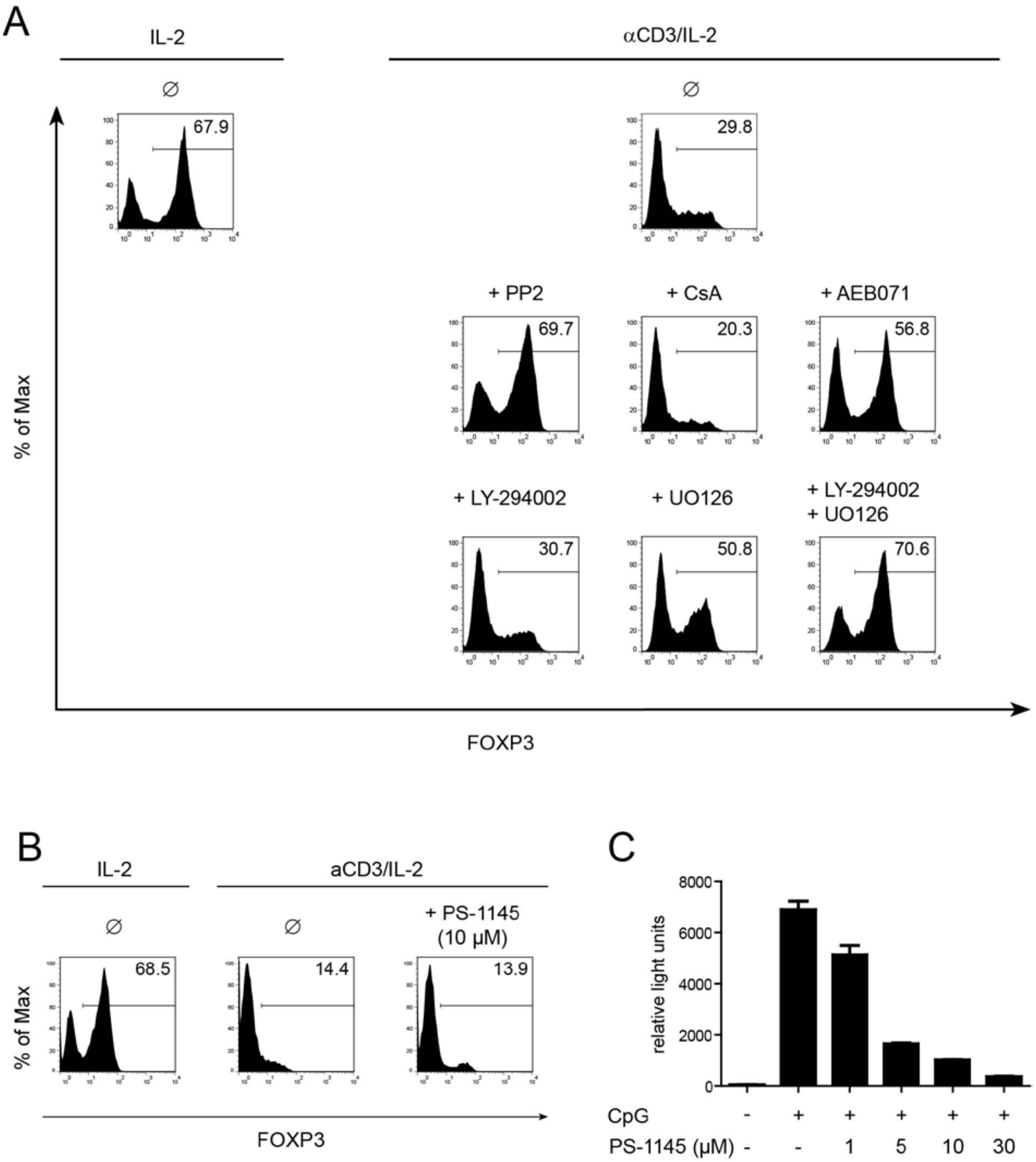

Supplementary Figure S2: **Intracellular pathways employed by the TCR to suppress Foxp3 expression. (A, B)** Intracellular Foxp3-staining of iTreg induced for 72 hr, washed and re-cultured for 48 hr in the presence of IL-2 with or without αCD3 and the following inhibitors, as indicated: PP2 (10 µM) against Src-Kinases, CsA (10 ng/ml) against Calcineurin, AEB071 (3 µM) against PKC, Ly294002 (10 µM) against PI3K and UO126 (10 µM) against MEK/ERK (all in A) or PS-1145 against NFκB (10 µM) (B). (C) Luciferase reporter assay of 293-mTLR9-luc cells cultured with or without 2 µM CpG for 24 hr in

absence or presence of different concentrations of PS-1145, used as a positive control for PS-1145 activity. Bars indicate the mean ± SD of triplicate determinations.

Supplementary Fig. 3

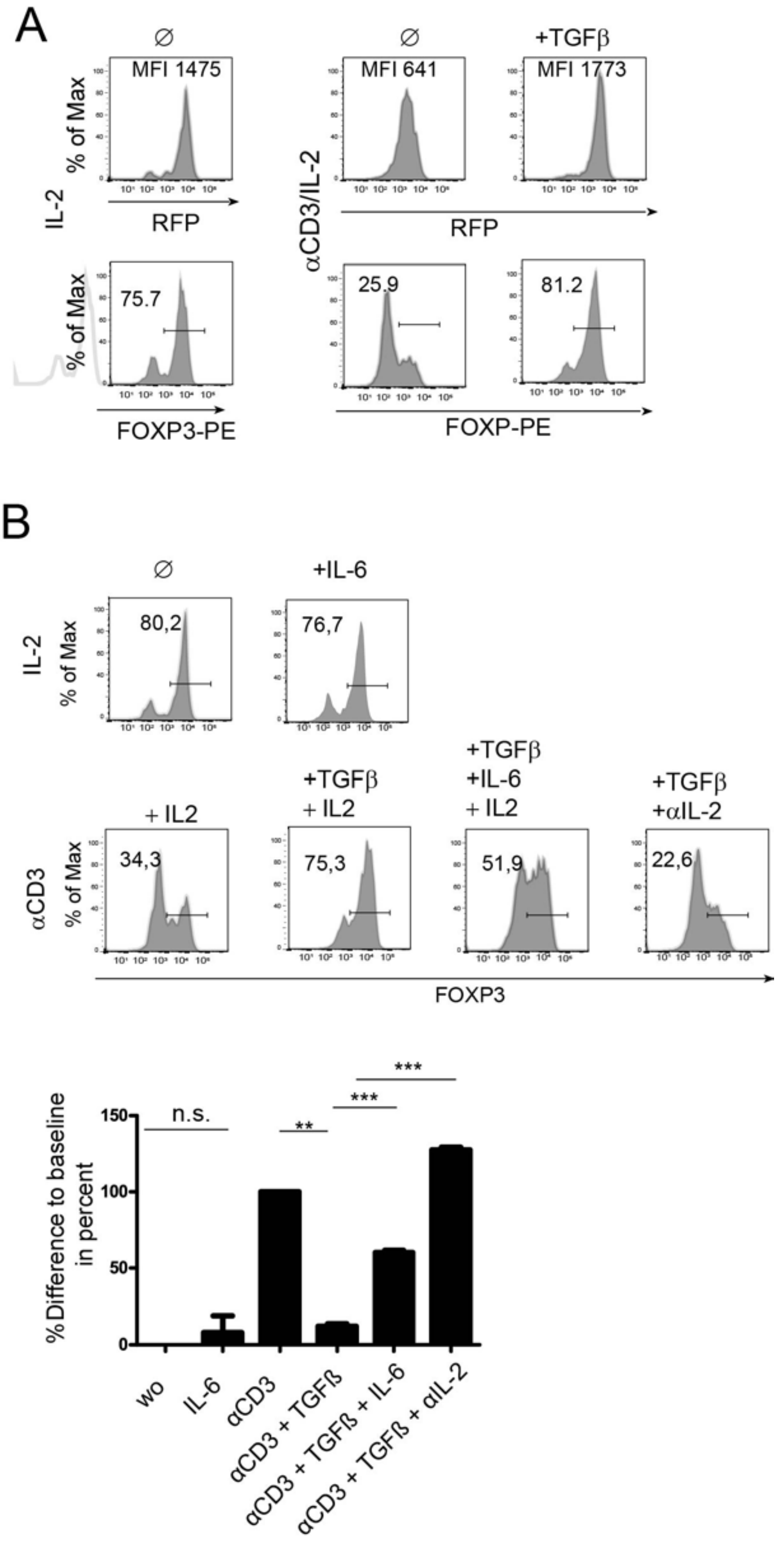

Supplementary Figure 3: **Confirmation of results in Fig. 2 using RFP reporter cells**. iTreg were induced from CD4+ FIR cells, rested for 48 hr with IL-2 and without αCD3, RFP+ cells were sorted and re-cultured with the indicated conditions for further 48 hr. RFP (A, top panels; mean fluorescence intensity, MFI) or FOXP3 expression (all other panels; numbers indiciate % of FOXP3+ cells) was determined. (A) Two experiments witrh similar outcome,

RFP purity after sorting was 98 and 99%. (B) Four experiments (histograms of one typical experiment and statistics (Student`s t-test) of all four). RFP purity after sorting was between 91 and 98%. Baseline represents cells re-cultured with IL-2 only. n.s.: not significant. **: p< 0.01; **

Supplementary Fig. 4

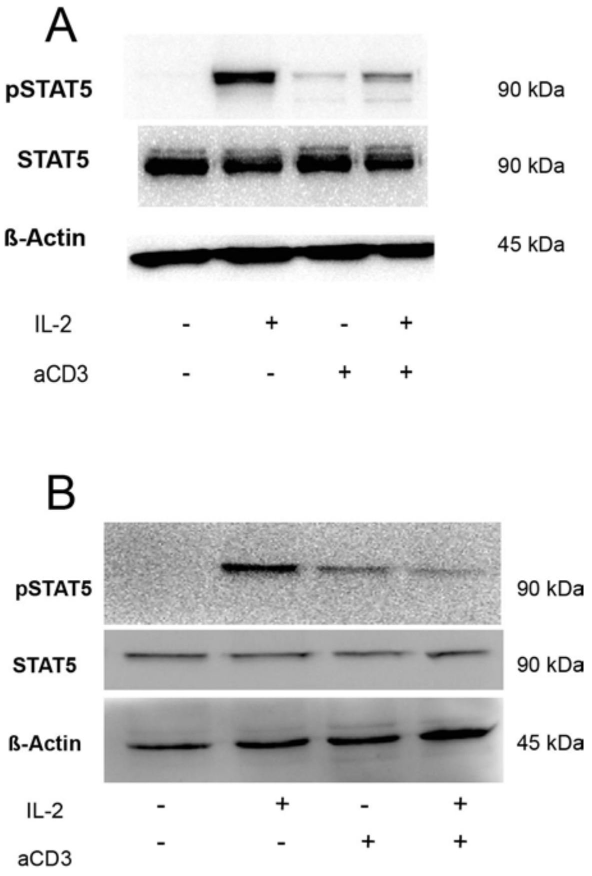

Supplementary Figure 4: **TCR-stimulation hampers IL-2 signalling.** (A,B) Western Blot for pSTAT5, STAT5 and β-Actin in lysates of iTregs re-cultured for 24 hr under the indicated conditions, deprived of IL-2 for 1h and resupplied with IL-2 (200 U/ml) for 10min. (A) iTregs from normal mice re-cultured immediately after induction. (B) RFP^{+} iTregs from FIR mice sorted (purity 99%) after 72 hr induction and 48 hr resting, then re-cultured. Four (A) and two (B) experiments with similar outcome.

Supplementary Fig. 5

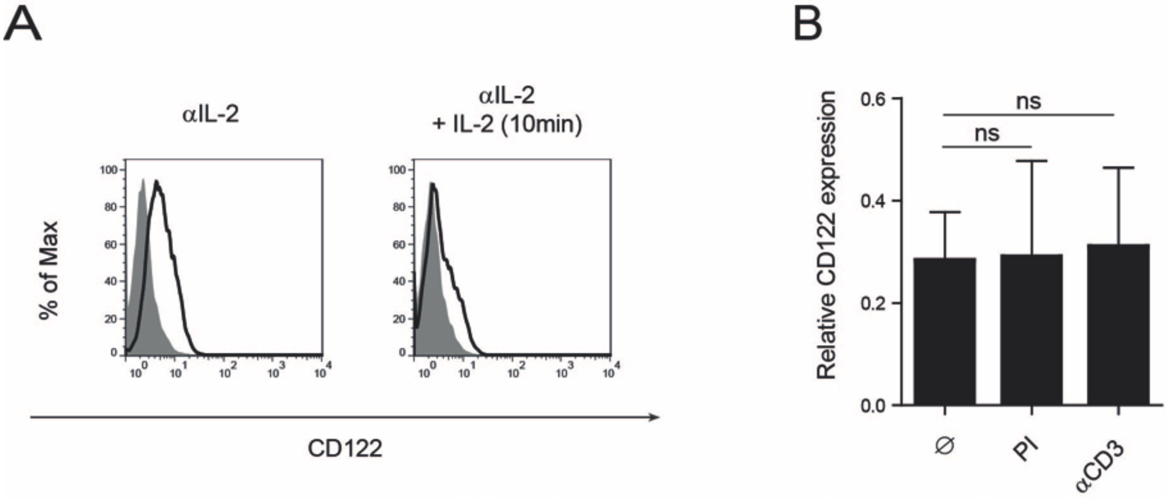

Supplementary Figure 5: **FACS analysis of CD122 is disturbed by I-2.** (A) An experiment similar to the one described in Fig. 3B was performed, however αIL-2 instead of IL-2 was added during re-culture and CD122 was stained with or without prior (10 min) addition of rh IL-2 (200 U ml^{-1}) into the staining tube. White area: anti-CD122, grey area: isotype control. (B) iTregs were re-cultured for 24 hr under the indicated conditions, cell lysates and total RNA were prepared and a qPCR for CD122 compared to HPRT was run using the primers 5' TTG AAC CAT ACT TGG AAT TTG AG 3' (Fwd), 5' GGA TCA GCA TCT CCA AGA AGA3' (Rev) and 5'-6-Fam-GCA TCC GTA TTA AGC CTC AAG-Tamra-3' (probe). Statistical evaluation plus SD (Student`s t-test) of three consecutive experiments.

Supplementary Fig. 6

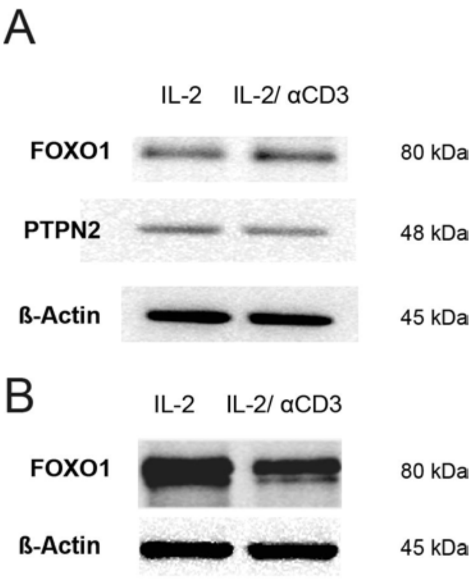

Supplementary Figure 6: **TCR stimulation does not influence amounts of PTPN2 and FOXO1 in nTreg, but influences FOXO1 in iTreg.** Western Blot for FOXO1 (A,B), PTPN2 (A) and β-Actin in lysates of sorted $CD4^+RFP^+$ nTreg (A; purity 98%) or iTreg, stimulated (A) or re-cultured (B) for 48 hr in the presence of IL-2 with or without αCD3. (A,B) Two experiments with similar outcome.

Supplementary Fig. 7

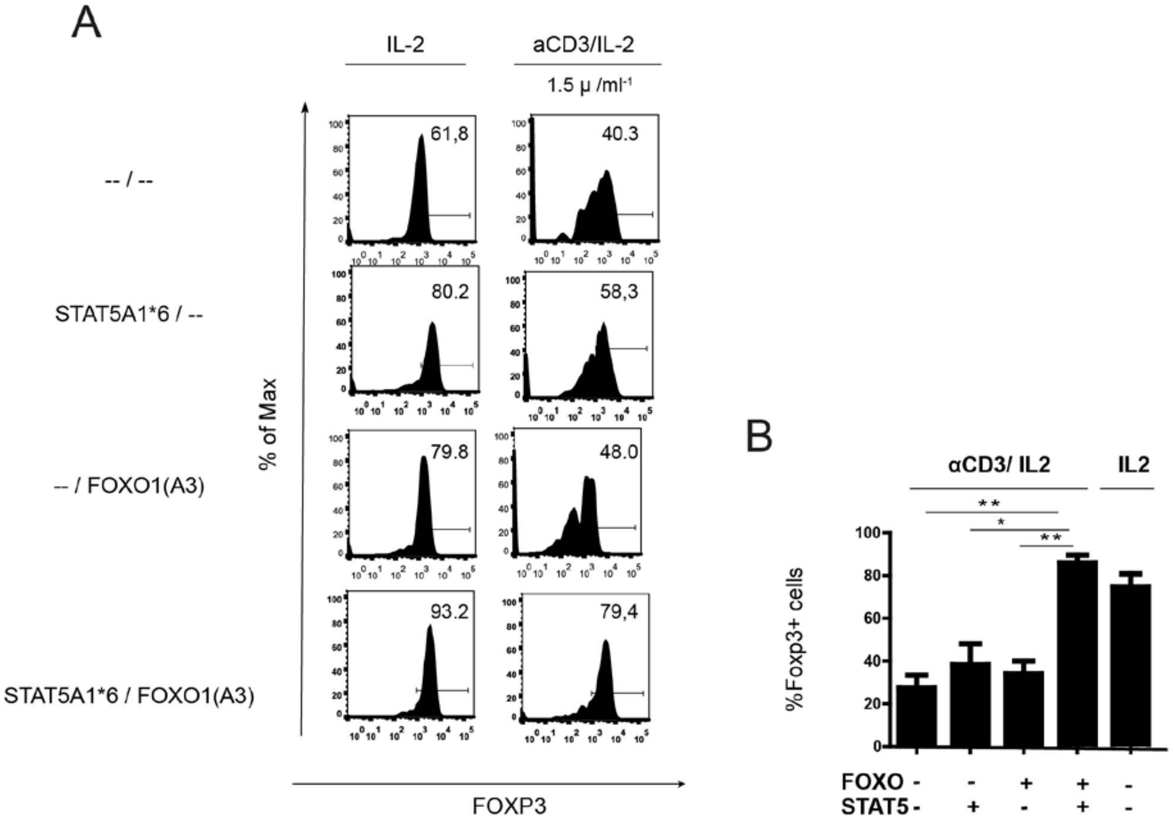

Supplementary Figure 7: **Overexpression of CA STAT5 and CA FOXO1 overcome the negative TCR-signal.** (A) Primed iTregs of FIR mice were retrovirally transduced twice within 48 hr with or without constitutively active (CA) STAT5 or CA FOXO1 as indicated, followed by sorting of RFP^+ cells (purity 96%) and 48 hr of re-culture under the indicated conditions. Intracellular staining of FOXP3 in cells gated according to the indicated infection status. Three experiments with similar outcome. Histograms from one representative experiment and summary (+/- SD) (student`s t-test) of all experiments. *, $P < 0.05$; **, $P < 0.01$.

Supplementary Fig. 8

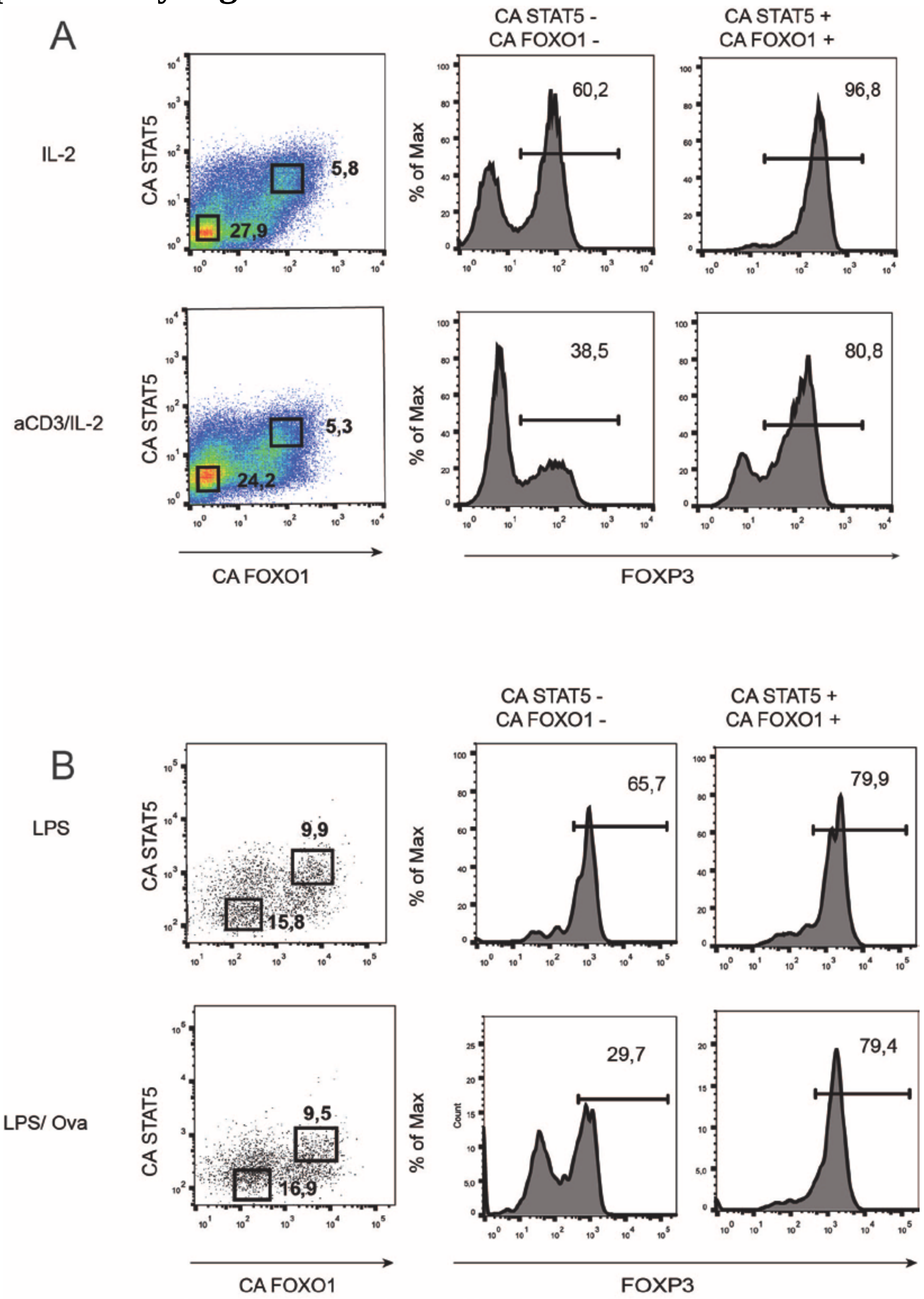

Supplementary Figure 8**. No relative growth advantage of Foxp3 negative cells. (**A) iTregs were infected with retroviruses, recultured for 48 h, as described for Fig. 6A, and analysed by flow cytometry. (B) OT II/FIR cells from the LN cells of one of the mice described in Fig. 7 are also depicted. (A,B) Numbers indicate frequencies of cells in the marked areas (respective left two panels) or of Foxp3$^+$ cells according to the indicated range gate setting (histograms). Representative of 5 experiments in (A) and of a total of 8 mice per treatment group analysed in two separate experiments in (B).

Supplementary Fig. 9

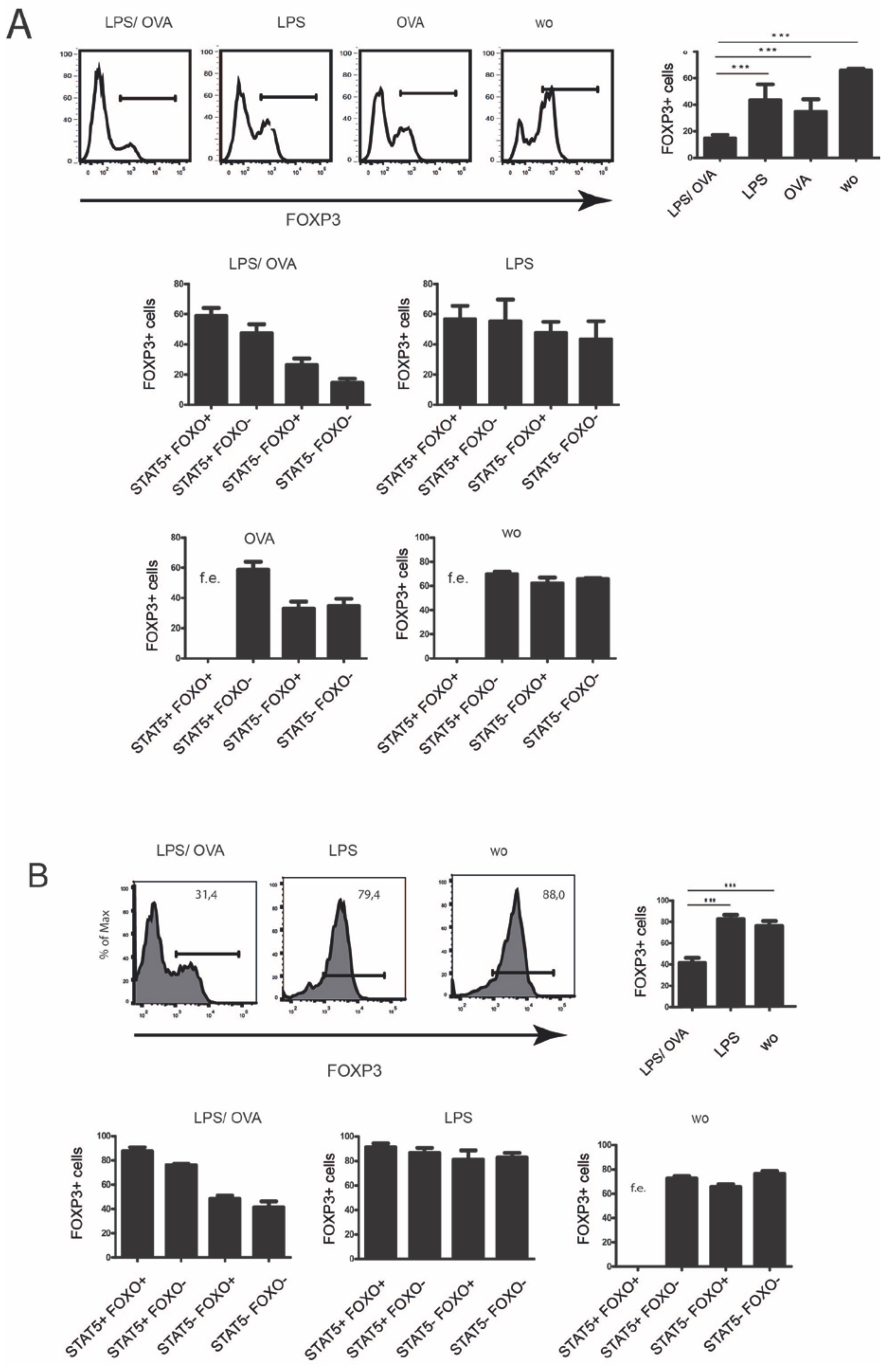

Supplementary Figure 9: **Antigen-specific downregulation of FOXP3 in iTregs *in vivo***. iTregs were induced from OT II (A) or from OT II/FIR (B) $CD4^+$ T cells and were transfected with either CA FOXO1 or CA STAT5 or both. OT II/FIR were then sorted for RFP^+ cells (purity 97%). OT II (86 % $Foxp3^+$) and sorted OT II/FIR cells were transferred i.p into C57BL/6 recipient mice with or without OVA and/or LPS (4 mice per group and experiment). Spleen cells were analysed 7 days later by flow cytometry, as described in Fig. 7. One experiment each for OT II and OT II/FIR cells. f.e.: few events. ***, P < 0.001.

Supplementary Fig 10

full size scans of the Western blot images shown in the manuscript figures:

A: Fig.3A; B: Fig.4A; C: Fig.4B

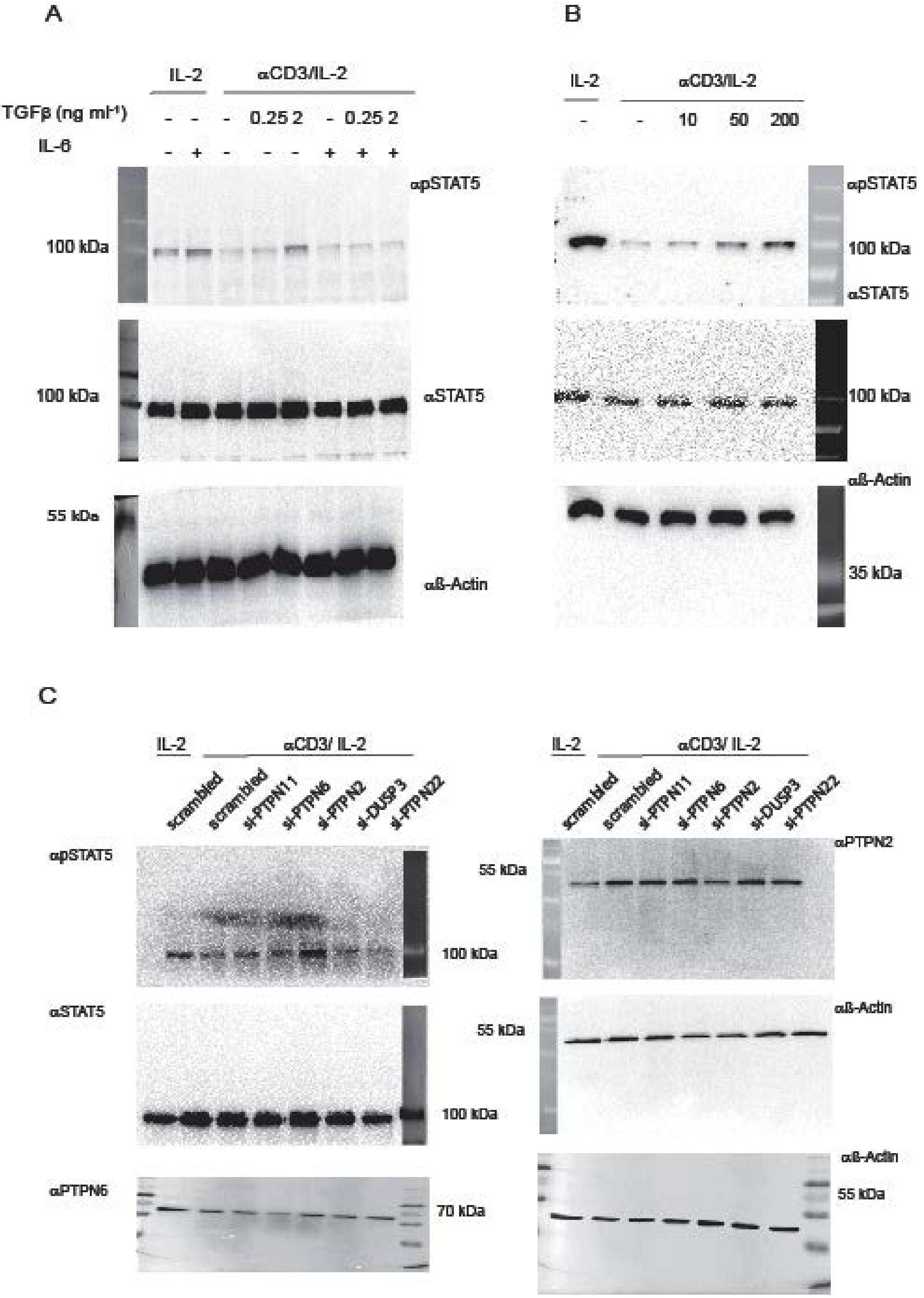

Supplementary Fig. 11

full size scans of the Western blot images shown in the manuscript figures:

A: Fig.4C; B: Fig.5; C: Fig.S4A; D: Fig.S4B.

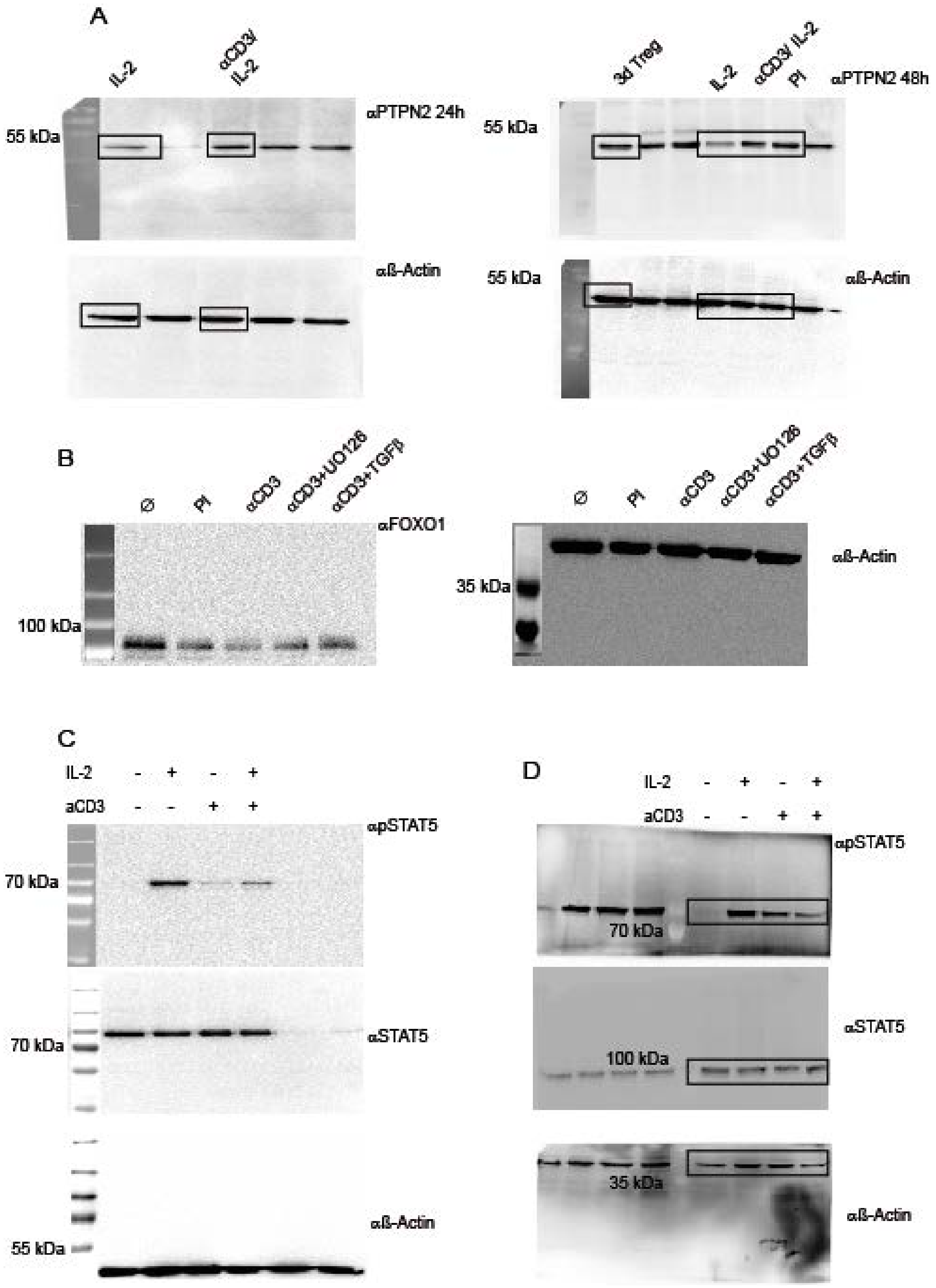

Supplementary Fig12

full size scans of the Western blot images shown in the manuscript figures:

A: Fig.S6A; B: SF6B.

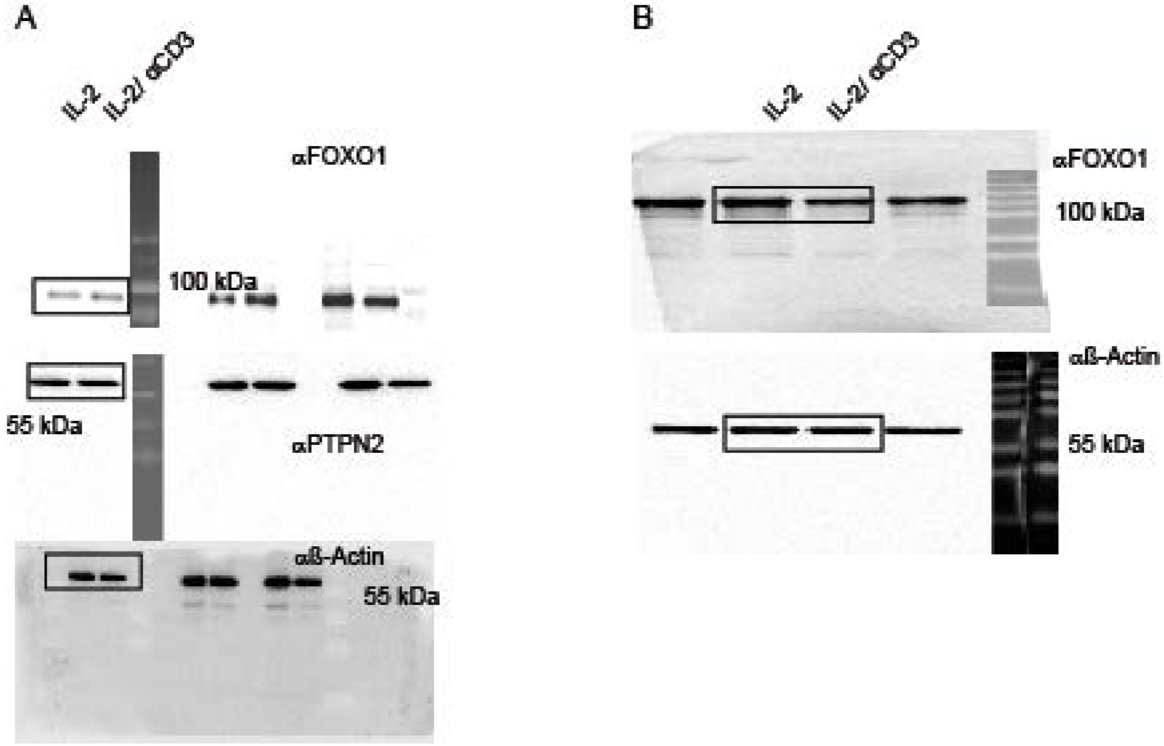

Supplement: Supplementary Information — Supplementary Figures 1-12 [file ncomms9576-s1.pdf]
